# Immune-Signatures for Lung Cancer Diagnostics: Evaluation of Protein Microarray Data Normalization Strategies

**DOI:** 10.3390/microarrays4020162

**Published:** 2015-04-02

**Authors:** Stefanie Brezina, Regina Soldo, Roman Kreuzhuber, Philipp Hofer, Andrea Gsur, Andreas Weinhaeusel

**Affiliations:** 1 Molecular Diagnostics, Health & Environment Department, AIT Austrian Institute of Technology GmbH, Muthgasse 11, 1190 Vienna, Austria; E-Mails: stefanie.brezina@meduniwien.ac.at (S.B.); regina.soldo@ait.ac.at (R.S.); roman.kreuzhuber@ait.ac.at (R.K.); 2 Department of Medicine I, Institute of Cancer Research, Comprehensive Cancer Center, Medical University Vienna, Borschkegasse 8a, 1090 Vienna, Austria; E-Mails: philipp.hofer@meduniwien.ac.at (P.H.); andrea.gsur@meduniwien.ac.at (A.G.)

**Keywords:** protein microarrays, cancer research, lung cancer, bioinformatics, biomarkers

## Abstract

New minimal invasive diagnostic methods for early detection of lung cancer are urgently needed. It is known that the immune system responds to tumors with production of tumor-autoantibodies. Protein microarrays are a suitable highly multiplexed platform for identification of autoantibody signatures against tumor-associated antigens (TAA). These microarrays can be probed using 0.1 mg immunoglobulin G (IgG), purified from 10 µL of plasma. We used a microarray comprising recombinant proteins derived from 15,417 cDNA clones for the screening of 100 lung cancer samples, including 25 samples of each main histological entity of lung cancer, and 100 controls. Since this number of samples cannot be processed at once, the resulting data showed non-biological variances due to “batch effects”. Our aim was to evaluate quantile normalization, “distance-weighted discrimination” (DWD), and “ComBat” for their effectiveness in data pre-processing for elucidating diagnostic immune-signatures. “ComBat” data adjustment outperformed the other methods and allowed us to identify classifiers for all lung cancer cases *versus* controls and small-cell, squamous cell, large-cell, and adenocarcinoma of the lung with an accuracy of 85%, 94%, 96%, 92%, and 83% (sensitivity of 0.85, 0.92, 0.96, 0.88, 0.83; specificity of 0.85, 0.96, 0.96, 0.96, 0.83), respectively. These promising data would be the basis for further validation using targeted autoantibody tests.

## 1. Introduction

Lung cancer is the leading cause of cancer-related death worldwide, responsible for around 1.59 million deaths in 2012 [1]. This disease is characterized by high heterogeneity in terms of pathological features and is commonly classified into two major groups, small-cell lung carcinoma (SCLC), accounting for approximately 15% of lung cancer cases, and non-small cell lung carcinoma (NSCLC). NSCLC includes mostly squamous cell carcinoma (SqLC), adenocarcinoma (AdCa), and large cell carcinoma (LCLC) [2,3]. Most lung cancers are detected at advanced stages, reducing the likelihood of cure. Five-year survival rates for all stages are about 15% [4]. One reason for late lung cancer detection is that usually lung cancer symptoms, such as cough, chest pain, and weight loss, do not appear until the disease is already in an advanced stage. Moreover, the lung is a visceral organ with a complex branching structure, which makes an entire examination impossible. Additionally, traditional diagnostic methods like computer tomography (CT) or X-ray often yield low positive predictive values (PPV), indicating that only few patients with a positive test result were confirmed with lung cancer after additional biopsy. This circumstance results in unsatisfactory performance of many diagnostic tests, high downstream costs and unpleasant procedures for the patients [5]. Therefore, it is important to find novel markers for early detection of lung cancer by means of minimal invasive biomarker screenings using material such as blood, sputum, or exhaled breath [6]. 

Today, it is known that proteins, expressed by tumors, often evoke a humoral immune response in cancer patients [7]. This response occurs because these tumor-associated antigens (TAAs) are mostly altered in some way. They may be unique to cancer and germ cells, found only in specific tumors, or may be mutated, misfolded, overexpressed, aberrantly degraded, or aberrantly glycosylated [8,9,10]. The response of the human immune system to such TAAs becomes apparent with the production of autoantibodies targeting these antigens [11]. This suggests that the immune system is able to detect aberrant structure, distribution, and function of certain proteins involved in tumorigenesis [12]. The finding that cancer patients produce autoantibodies against tumor antigens encourages the idea that these autoantibodies could facilitate cancer diagnosis and prognosis [10,11,13,14]. Moreover, the relevance of autoantibody-based biomarkers is emphasized by the fact that autoantibodies against TAAs can be found up to five years before tumors become symptomatic or clinically detectable with conventional methods [7,15,16,17,18,19]. 

Autoantibodies have been detected in several human cancers, but only a few antigen-autoantibody reactivities have made their way into clinical practice. One reason may be that most autoantibodies occur only in a subset of cancer patients, resulting in low sensitivity of single antigens as tumor biomarkers [11,20]. This lack of sensitivity indicates that a single tumor autoantibody will not be sufficient as biomarker [21]. Therefore, biomarker panels, including cancer-specific autoantibodies are considered as promising tools for early diagnosis and prognosis of cancer. For this purpose, autoantibody signatures, the molecular finger print of antibodies in a certain disease, have to be identified [22]. 

For identification of potential TAAs, large screening sets are required [23]. A highly multiplexed approach, like protein microarrays, offers a suitable platform for autoantibody marker discovery while using only small amounts of serum or plasma samples [24,25]. This technology enables screening of humoral response against thousands of potential TAAs in order to identify novel biomarker panels for cancer diagnosis [23]. For this purpose, antigens, which are either derived from recombinant protein expression or are isolated from tumor cell lines, are immobilized and their reactivity with sera from patients is investigated [26].

High-dimensional microarray data require careful statistical handling. In general, the *first step* is log2 transformation of the data in order to achieve symmetric ratios of fold-changes, independent from increase or decrease in intensity values [27]. The *second step* requires adjustment of the data for systematic biases. Many microarray studies are affected by batch effects, which can often not be avoided, because not all samples can be processed in one single batch [28]. This introduces non-biological differences, which impede comparability of the processed samples. Combination of microarray data derived from different experimental batches enables increasing statistical power of microarray studies. Therefore, adjustment for batch effects has to be performed [28,29,30,31]. One method is quantile normalization, which aims to adjust the distribution of probe intensities for each array in the data set to the same level [30]. To minimize batch effects in data sets derived from multiple experiments, algorithms, such as the empirical Bayes method referred to as “combating batch effects when combining batches of gene expression microarray data” (ComBat) [31] or “distance-weighted discrimination” (DWD) [32], which have been proposed to be effective at adjustment for systematic biases, can be applied. Batch effects as well as batch effect removal methods are often investigated visually by means of principal component analysis (PCA) plots [33]. A novel method combining PCA and variance component analysis (VCA) is able to evaluate the contribution of certain sources to the variance in the microarray data set. This approach is referred to as principal variance components analysis (PVCA) [28,34]. The *third step* after data pre-processing is comprehensive analysis of the microarray data set. This can be done by means of the software Biometric Research Branch BRB-ArrayTools, developed by Richard Simon. This software includes a broad variety of methods for predictive classifier development. Supervised machine learning methods, such as class comparison and class prediction, are available with complete cross-validation [35]. The aim of class comparison analysis of DNA microarrays is the *univariate* identification of differentially expressed genes in two different specimen phenotypes [36]. The same statistical analysis methods which are usually used for gene expression studies were applied to our protein microarray data. When using protein microarrays for the identification of tumor autoantibody signatures, class comparison analysis enables determination of differentially reactive antigens with autoantibodies of cancer patients compared to controls [37]. Another method included in this software is class prediction. This method aims at developing a *multivariate* statistical model to predict the class of a sample based on its signal pattern on the microarray. For the development of such models, features that enable discriminating the predefined classes have to be identified and accuracy of the built predictor is estimated [38]. For this purpose, it is advisable to use complete cross-validation which includes feature selection and model development. By doing so, overfitting of the model to the used data set is avoided in contrast to incomplete cross-validation, which does not include feature selection in the process of cross-validation, or no cross-validation. Another advantage of cross-validation, especially leave-one-out cross validation (LOOCV), is that small data sets are used efficiently. Each sample is withheld once during feature selection and model development and is then classified based on the developed model. This process is iterated for each sample [39]. Analysis of microarray experiments enables the detection of features which are correlated to a phenotype or to find a predictor or classifier to predict the phenotype of a new sample [40]. These predictors could be applied in clinical diagnostic testing, for assessment of prognosis, or treatment decision [39].

In the present study, we used a high-density protein microarray for the detection of autoantibody signatures in lung cancer. This protein microarray was processed with purified immunoglobulin G (IgG) from 100 lung cancer cases and 100 matched lung cancer-free controls. The lung cancer group comprised 25 samples from each of the four main histological lung cancer types. Since the sample cohort of this study included 200 different samples, not all samples could be processed at once. Therefore, samples belonging to one histological lung cancer entity and their matched controls (results in a total of n = 50) had to be processed in two different runs. As already mentioned, adjustment for batch effects has to be done when combining data from different microarray experiments [41]. For this purpose, different normalization and batch effect adjustment methods were used, including quantile normalization, DWD, and ComBat. Currently a great number of normalization algorithms and data transformation methods are available and there are several publications comparing the performance of some of these [28,30,41,42]. However, it is still difficult to decide which method performs best for a certain data set. Moreover, it should be kept in mind that analysis results are strongly influenced by the applied normalization method [27,42]. 

We aimed at comparing different normalization and data adjustment methods in terms of effectiveness and their influence on subsequent data analysis. For this purpose, principal component analysis (PCA) was used to investigate the variance in the data set before and after applying different data pre-processing methods. Furthermore, overlaps of significant antigens derived from class comparison were compared between different sample cohorts with all normalization methods. Since the study was focusing on the identification of a tumor-autoantibody-based biomarker panel for early diagnosis of lung cancer, the development of multivariate classifiers for each histological type of lung cancer and for the combination of all lung cancer types was done by means of class prediction. This was performed with quantile normalization and ComBat adjustment. Classifier panels identified during this study are already patented [43]. 

## 2. Experimental Section 

### 2.1. Patient Cohort

The study population comprised 100 newly diagnosed and previously untreated lung cancer patients and 100 lung cancer-free control samples. All samples used in the present study were provided by the biobank of the Molecular Epidemiology group headed by Andrea Gsur at the Medical University, Department of Medicine I, Institute of Cancer Research, Vienna. Cases were recruited from two pulmonological centers in Vienna and Lower Austria. Regarding histological data, cases were further classified as 25 small-cell lung carcinoma (SCLC), 25 squamous cell carcinoma (SqLC), 25 large cell carcinoma (LCLC), and 25 adenocarcinoma (AdCa). Seventy-two hospital-based controls were derived from the Department of Orthopedics of the Medical University of Vienna. Only subjects with negative chest radiography and no history of malignant disease were selected as controls. Additionally, 28 controls were recruited from the colorectal cancer screening project “Burgenland Prevention Trial of Colorectal Cancer Disease with Immunological Testing” (B-PREDICT). These individuals underwent colonoscopy and did not show any pathological finding. All participants were Austrian Caucasians. Clinical characteristics of the samples are summarized in Table 1. 

Blood was collected using Vacuette BD vacutainer^®^ tubes (Greiner Bio-One, Kremsmünster, Austria) with sodium heparin as anticoagulant. After collection, heparinized blood was centrifuged at 2000 × *g* for 10 minutes and plasma was removed from the cell pellet and stored at −80 °C. Written informed consent was obtained from all participants and research protocols were approved by the institutional review boards.

**Table 1:** Clinical characteristics of the study population.

Variable		Lung cancer cases (n = 100)				controls (n = 100)
		SCLC (n = 25)	SqLC (n = 25)	LCLC (n = 25)	AdCa (n = 25)	
Age^a^ (years, mean, range)		65.0 (41–85)	61.9 (42–80)	62.6 (40–81)	63.0 (39–79)	61.9 (33–89)
Sex						
	male	18	21	13	17	69
	female	7	4	12	8	31
Smoking						
	never	1	0	2	3	24
	former	13	10	12	14	42
	current	11	15	11	8	34
Clinical tumor stage						
	IA		4	4	6	
	IB	1				
	IIA	1	5			
	IIB	6	1			
	IIIA		5	5	4	
	IIIB		4	2	1	
	IV	10	2	11	8	
	n.a.	7	4	3	6	
lymph node metastasis		18	14	16	13	

^a^ At time of diagnosis.

### 2.2. Human IgG Isolation

Human immunoglobulin G (IgG) was isolated from plasma samples using the Melon^®^Gel Spin Plate Kit for IgG Screening (Thermo Scientific, Rockford, IL, USA) following the manufacturer’s instructions. IgG samples were stored over night at 4 °C until further processing. Concentration of purified IgG was measured spectrophotometrically at 280 nm on an Epoch Microplate Spectrophotometer (Biotek, Winooski, VT, USA). As blank sample Melon Gel Purification Buffer was used. Samples were measured in duplicates and arithmetic average was calculated. Pre-casted NuPAGE^®^ 4%–12% Bis-Tris polyacrylamide gels (Novex^®^, Carlsbad, CA, USA) were used to verify IgG purity. The strongest band was observed at 150 kDa, which corresponds to the molecular weight of undegraded human IgG [44]. Integrity of all samples was confirmed.

### 2.3. 16k Protein Microarray

The in-house printed 16k protein array comprised 5449 recombinantly expressed proteins derived from 15,417 cDNA clones, printed in duplicates on the microarray resulting in approximately 32,000 spots. An illustrated microarray design of the 16k array can be found in the supplementary section (Supplementary Figure S1). The cDNA clones are included in the UniPEx expression library purchased from ImaGenes GmbH (Berlin, Germany). Sequences included in this library comprise clones derived from human fetal brain, T-cells, lung, and colon protein expression libraries. The library consists of clones with two differently modified pQE vectors. Protein expression from clones carrying the pQE80LSN vector is induced by isopropyl-β-D-thiogalactopyranosid (IPTG). Clones with the pQE30NST vector inserted exhibit auto-induced protein expression. Both types of vectors include an N-terminal 6xHis-tag [45]. Protein expression and purification was conducted as described in Stempfer *et al.* [22]. To estimate purity of the UniPEx proteins after purification with Ni-NTA, protein eluates were run on SDS-PAGE using NuPAGE^®^ Novex 4%–12% Bis-Tris gels (Supplementary Figure S2).

SU8 epoxide-coated glass slides were printed with contact printing technology using a NanoPrint^TM^ LM210 (Dynamic Devices, Wilmington, DE, USA) with 48 SMP2 Stealth Microarray Printing Pins (TeleChem ArrayIt Microarray Division, Sunnyvale, CA, USA). *E. coli* lysate in the concentrations 0.3, 0.4, and 0.5 mg mL^−1^ was used as positive control (n = 281), and elution buffer (50 mM Na2HPO4, 500 mM imidazole, 0.3 M NaCl, 0.01% SDS and 0.005% NaN3) was spotted as negative control (n = 82) onto the slides. Printed protein slides were stored at 4°C protected from light until processing.

### 2.4. Protein Microarray Processing

Protein microarrays were blocked for 30 minutes with DIG Easy Hyb solution (Roche, Basel, Switzerland), washed three times for 5 minutes with 1 × PBS + 0.1% Triton X buffer (PBS 10× pH 7.4 (Life Technologies, Carlsbad, CA, USA), Triton X-100 (Sigma-Aldrich, St. Louis, MO, USA) and rinsed with ddH_2_O. Slides were dried for 2 minutes by centrifugation at 900 rpm. Purified IgG samples were adjusted to a concentration of 0.4 μg μL^−1^ with Melon Gel Purification Buffer and were finally diluted 1:2 with 2 × PBS + 0.2% Triton X buffer containing 6% milk powder. 490 μL of the diluted samples were applied onto a single-chamber hybridization gasket slide (Agilent Technologies, Santa Clara, CA, USA) and the 16k protein slide was placed on top, allowing the spotted protein side of the slide to face the IgG sample. Samples were incubated on the 16k microarray slides for 4 hours under rotating conditions, 12 rpm at room temperature. Slides were washed three times with 1 × PBS + 0.1% Triton X wash buffer and rinsed with ddH_2_O. As detection antibody, Alexa Fluor® 647 Goat Anti-Human IgG (H+L) (Molecular Probes^®^, Carlsbad, CA, USA) was used, diluted 1:10,000 in PBS + 0.1% Triton X-100 + 3% milk powder. Incubation was done for 1 hour. Slides were again washed three times with 1 × PBS + 0.1% Triton X washing buffer, rinsed with ddH_2_O and spin-dried. 

Microarray slides were scanned with an Agilent G2565CA Microarray Scanner System (Agilent Technologies, Santa Clara, CA, USA) using a helium-neon laser (633 nm, red dye channel). Slides were scanned with a resolution (pixel size) of 10 μm and sensitivity level of the red channel photomultiplier tube (PMT) was set to 70%. After scanning, images of slides were loaded into the GenePix^®^ Pro 6.0 software (Molecular Devices, Sunnyvale, CA, USA) for subsequent data extraction. Median fluorescent intensity, corrected for local fluorescent background, for each IgG-probed protein spot was exported to a .gpr file for further statistical analysis. 

### 2.5. Data Processing

Statistical analysis of the microarray data was performed using the R-based package BRB-ArrayTools, developed by Richard Simon [35]. Background-corrected median fluorescence values were loaded into the program and were log2-transformed. Furthermore, an average over replicate spots was calculated since all proteins were printed in duplicates resulting in a microarray comprising approximately 32,000 features. The intensity minimum threshold was set to a value of 100. If a feature was flagged in at least 50% of the arrays, it was excluded from analysis. 

Data was normalized using different approaches and was further subjected to different statistical analyses. One method was quantile normalization, which equals distribution of values among different arrays. The second approach was data transformation using “Combating batch effects when combining batches of gene expression microarray data” (ComBat) in order to adjust data for batch effects [46]. For the application of ComBat, unnormalized data was used and missing values, which were due to flagging of low-quality features, were imputed. This was done using the package ‘impute’ for imputation of missing microarray data in R [47]. The third approach was distance weighted discrimination (DWD) which adjusts for systematic microarray data biases [32]. By means of using DWD, unnormalized data is loaded into the software but only two batches can be merged at one time. Therefore, a stepwise approach was applied for data adjustment. According to the PCA plot of the unnormalized data (Figure 3), two main groups of batches can be observed in the raw data. Therefore, the runs of each group were merged first before merging the two main groups in the last step. A similar approach was used by Benito and colleagues [32]. PCA [48] and PVCA [49] were used to investigate batch effects and underlying sources for variation.

Further statistical analysis was performed with BRB-ArrayTools. Mainly class prediction with complete leave-one-out cross-validation (LOOCV), which includes the steps of feature selection and classification model development, and class comparison were applied to different data subsets, which were pre-processed by different methods. Analysis was performed for all lung cancer cases *versus* all controls as well as for distinct histological types of lung cancer and their matched controls. Class comparison analysis was performed with a significance threshold of *p* < 0.001, resulting in a list of significant differentially reactive antigens when comparing two sample groups. These lists were generated with unnormalized, quantile normalized, DWD-adjusted, and ComBat-adjusted data. Besides analysis of the sample groups, including all lung cancer samples *versus* all controls and the groups comprising only the histological subtype of lung cancer *versus* statistically matched controls, class comparison analysis was applied on the data of each histological subtype derived from single processing batches (run 1 to run 4). Moreover, cross-run class comparison was performed for each histological entity. The resulting lists of significant antigens were compared to each other for overlaps. 

Class prediction analysis was performed on the same data set including the analysis of all lung cancer cases *versus* all controls with quantile normalization and ComBat adjustment, as well as the analysis of distinct histological entities derived from multiple runs *versus* matched controls using quantile normalization, ComBat, and DWD adjustment. BRB-ArrayTools provides this supervised machine learning method with LOOCV. As feature selection methods, the greedy-pairs method [46] and the recursive feature elimination (RFE) method [50] were used. Methods used for model development were diagonal linear discriminant analysis, compound covariate predictor, nearest neighbor classification, nearest centroid classification, support vector machines and Bayesian compound covariate predictor [35].

## 3. Results

**Figure 1:**
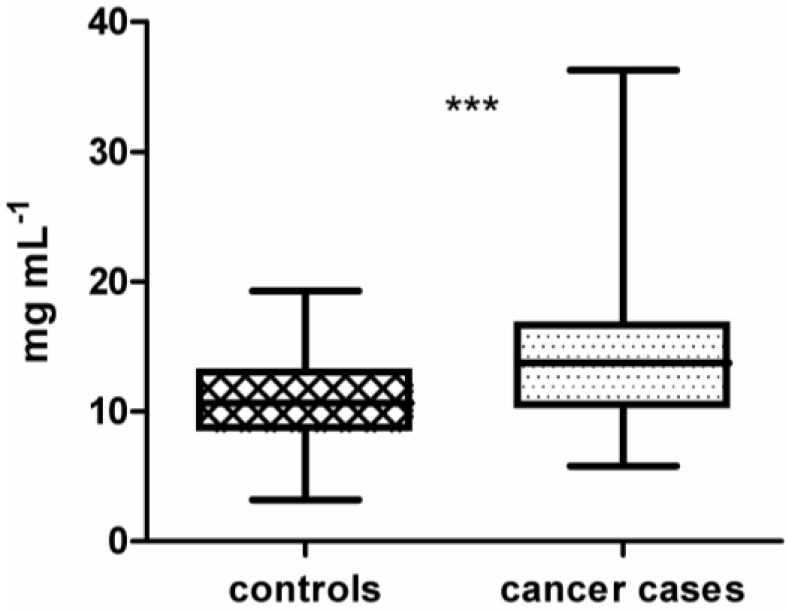
Boxplot of immunoglobulin G (IgG) concentrations from plasma of 100 controls (10.9 mg mL^−1^ ± 0.33) compared to 100 lung cancer cases (14.1 mg mL^−1^ ± 0.47). IgG concentrations of cases and controls are significantly different (unpaired *t*-test, *p* < 0.0001). Significance is indicated by asterisks (*** for *p*-value <0.001).

**Figure 2:**
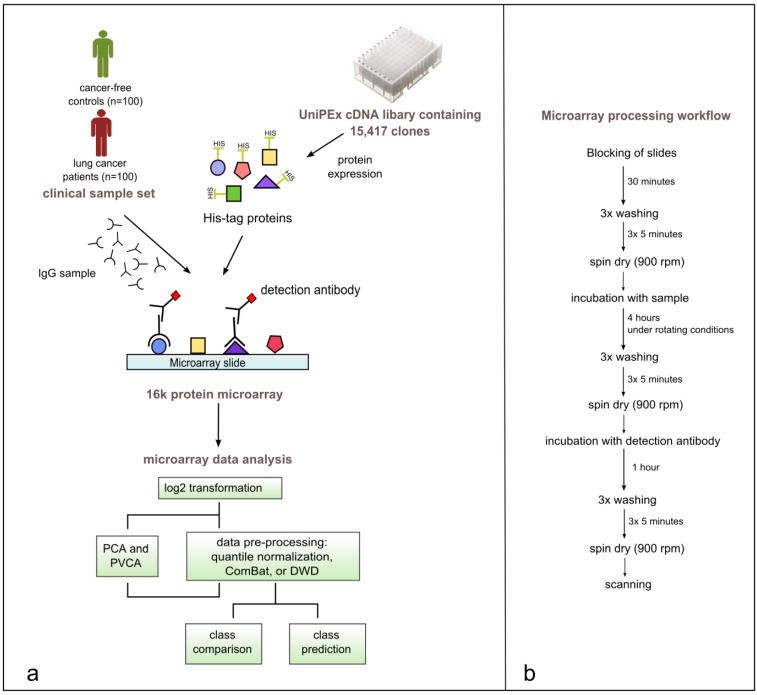
(**a**) Study workflow including the production of 16k protein microarrays, array processing with 200 clinical samples, data pre-processing, data visualization, and statistical analysis of microarray data using BRB-ArrayTools. (**b**) Illustrated microarray processing workflow.

In this study, samples from 100 lung cancer patients and 100 lung cancer-free controls were processed on the 16k protein microarray comprising 5449 recombinantly expressed human proteins derived from 15,417 cDNA clones. These proteins served as potential antigens and therefore capture molecules for tumor-specific autoantibodies from plasma. Purified IgG samples isolated from plasma were used for array processing. Preliminary results, obtained during development and optimization of the 16k protein microarray, showed that binding of non-IgG serum proteins to the microarray surface is influencing the specificity and reproducibility of the assay [51]. As depicted in Figure 1, across all isolated IgG samples the median concentration of IgG isolated from cancer cases (14.1 mg mL^−1^ ± 0.47) is significantly different from the median concentration of IgG isolated from lung cancer-free controls (10.9 mg mL^−1^ ± 0.33) (*p* < 0.0001 using unpaired *t*-test). To identify a set of antigens representative for the lung cancer-specific humoral immune response in cancer patients, intensity data gained from array images were analyzed using BRB-ArrayTools. The workflow of this study is shown in Figure 2.

The sample set (n = 200) was processed in six distinct runs, each including up to 36 samples. Cases and controls included in one run were statistically matched for sex, age, and smoking-habits. In the first four runs, each run included only one histological cancer subtype matched with hospital-based controls. Run 5 and 6 included cases from two subtypes matched with selected controls. 

To check the quality of the spotting process, each slide was scanned before sample processing and slides showing irregularities were excluded from sample analysis. Furthermore, we used an anti-Penta-His Alexa Fluor 532-conjugated antibody (Qiagen, Hilden, Germany) for processing of slides derived from different printing batches to investigate reproducibility of spotting. Correlation of log2 intensities of the detected His-tag proteins is in average 0.93 when comparing slides of different printing batches.

A preliminary test, which included samples from three different individuals, was performed using purified IgG from the same IgG purification preparation. Each specimen was processed in three replicates on the same day and in three replicates on the consecutive day. Correlation between the replicates was calculated. Pearson’s correlation coefficient for intra-experiment replicates was in average 0.96 and for inter-experiment replicates 0.95. Furthermore, the intra-experiment median coefficient of variation (CV) ranges from 0.20% to 0.63% and the inter-experiment median CV ranges from 0.53% to 1.00% (Supplementary Figure S3). CV values were calculated for each protein of the array. 

Additionally to this preliminary experiment, one run of samples had to be repeated due to quality irregularities of the 16k slides. The best 17 slides from these repeatedly processed slides were used to investigate the reproducibility of the samples when using a different IgG purification preparation and different batches of spotted microarrays. Although the samples were processed on slides with a lower quality, resulting Pearson’s correlation coefficients range from 0.79 to 0.93 for log2-transformed raw data with an average of r = 0.86, and between 0.96 and 0.99 with an average of r = 0.98 upon removal of batch effects with ComBat. For further statistical analysis the replicates of samples were not included. 

On the 16k protein microarray, each protein was printed in duplicates. Duplicate spots show a very high correlation of signals within an array. We calculated the correlation between duplicate spots within 38 arrays and obtained an average Pearson’s correlation coefficient of 0.98. This high correlation indicates that duplicate spots are sufficient to ensure reproducibility of the measured signals. 

As positive controls on the 16k protein microarray different concentrations of *E. coli* lysate (0.3 mg mL^−1^, 0.4 mg mL^−1^, and 0.5 mg mL^−1^) were used. Fluorescent intensities of these control spots increase in a linear manner with increasing concentration, resulting in an average coefficient of determination of 0.97 over all processed samples. With respect to array processing with purified IgG in different concentrations, it was shown that fluorescent intensity increases in a linear manner with increasing IgG concentration [51].

**Table 2:** Experimental design. Each run indicates one batch of samples, which was processed in one day. In total, all 200 samples of the sample set were processed in six distinct runs.

n	cases				controls	total
	SCLC	SqLC	LCLC	AdCa		
run 1	18				18	36
run 2		18			18	36
run 3			18		18	36
run 4				18	18	36
run 5	7	7			14	28
run 6			7	7	14	28
∑	25	25	25	25	100	200

Since our protein microarray data is derived from different processing batches, systematic variation in the merged data would affect subsequent data analysis. Therefore, three different normalization or data adjustment strategies were applied on the data set in order to investigate the effect of these methods on the analysis results. After normalization of the data, principal component analysis was used to investigate the properties of the data set. Additionally, class comparison was used to identify significant antigens, which are differentially reactive to autoantibodies of lung cancer patients compared to lung cancer-free controls.

### 3.1. Principal Component Analysis and Principal Variance Components Analysis

**Figure 3:**
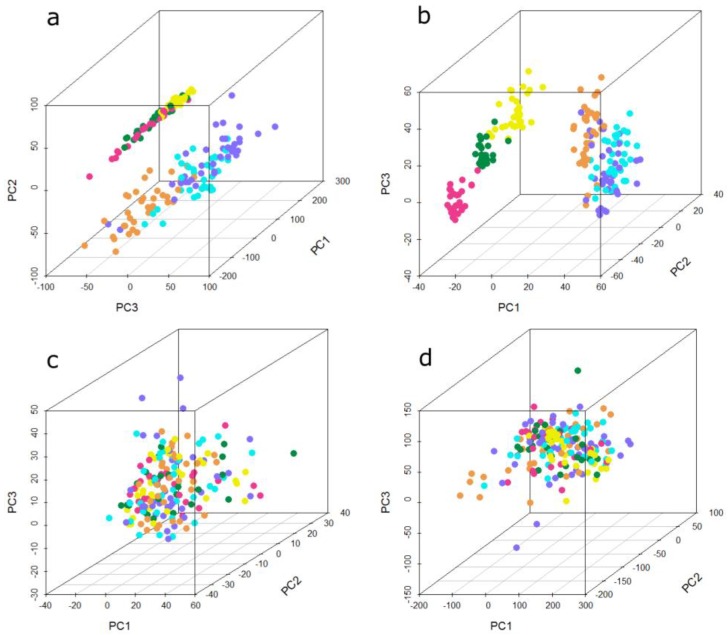
Principal component analysis (PCA) plot showing the first three principal components of the microarray data set (**a**) unnormalized, (**b**) quantile normalized, (**c**) ComBat-adjusted, and (**d**) DWD-adjusted. Each sphere represents one sample; the color of the sphere indicates the different experimental runs. Run 1 to run 6 is represented by the following colors: run 1 = orange, run 2 = cyan, run 3 = dark blue, run 4 = yellow, run 5 = dark green, run 6 = pink.

High-dimensional microarray data can be visualized by the reduction of dimensions by means of principal component analysis (PCA). The principal components, representing directions of the data with maximal variation, reveal similarities and differences between samples [52]. Figure 3 shows the first three principal components of the present microarray data set. Each sphere represents one sample and its color indicates the batch of sample processing, referred to as “run”. In the unnormalized data (Figure 3a), two groups of samples can be observed. The first three runs cluster into one group, the second group consists of the remaining three runs. One group consists of samples from three different histological types of lung cancer and their statistically matched controls, whereas the second group comprises all four used histological types of lung cancer and their matched controls, meaning that in both groups, three of the four used histological types are present. The PCA plot of the quantile normalized data is depicted in Figure 3b. This normalization method does not change the grouping of the batches compared to unnormalized data. The separation between the distinct runs in each group is even more obvious, especially between run 1, run 2, and run 3. By means of data adjustment with DWD (Figure 3c) and ComBat (Figure 3d), the previously observed groups become dispersed. In particular the ComBat algorithm achieves a more even distribution of the batches, whereas with DWD an accumulation of most samples in the center surrounded by a few samples outside of this center can be observed. 

**Figure 4:**
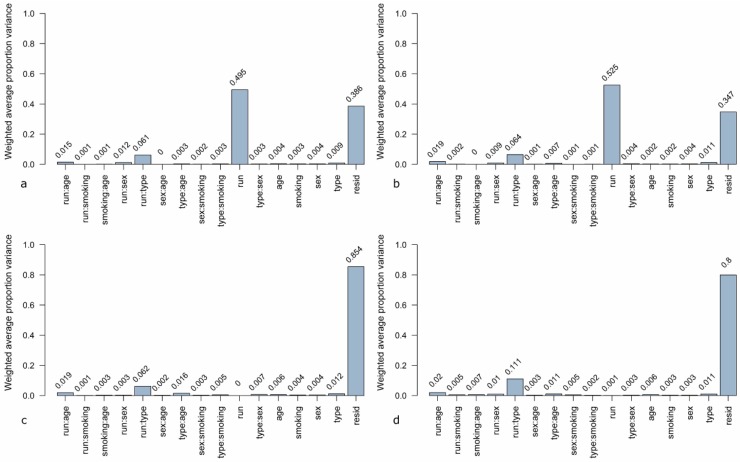
Principal variance components analysis (PVCA) of the microarray data set (**a**) unnormalized, (**b**) quantile normalized, (**c**) ComBat-adjusted, and (**d**) DWD-adjusted. Contribution to variance was estimated for the following factors: run = experimental runs (run 1 to run 6), type = sample type (cancer or control), sex = female/male, age = age group (0–56, 56–64, 64–70, 70–100 years), smoking = smoking habit (current, never, former smoker), resid = residual weighted average proportion variance. Single factors were investigated as well as combinations of factors.

A novel method for estimation of sources for variability in microarray data is principal variance components analysis (PVCA). In this analysis, a threshold for the percentage of variability is defined and the number of principal components representing this percentage is included in the further steps [34]. Here, the threshold was set to 60%. The defined principal components (PCs) are matched to known sources of variation by variance component analysis (VCA). The resulting weighted proportion of variance shows the proportional effect of each source on the data set [28]. The PVCA analysis of the unnormalized data (Figure 4a) shows that the highest contribution (49.5%) to variation is due to the experimental run. The second highest contribution is due to undefined residual effects (38.6%) which represent the variance in the data set which cannot be explained by known factors. The third most weighted variance is contributed by the combination of experimental run and sample type (lung cancer case or control). When using quantile normalization (Figure 4b), the contribution of the experimental run increases to 52.5%. This effect can be reduced to 0% when using ComBat (Figure 4c) and to 0.001% by means of DWD (Figure 4d). The combination of the effects “experimental run” and “sample type” remain similar when using quantile normalization and ComBat, only with DWD a slight increase to 1.11% can be observed. Other factors, like age, sex, and smoking habit, do not have any effects (0.02%–0.06%) independent of the data pre-processing method. The sample type (lung cancer case or cancer-free control) has just a slightly higher effect on variance in each data set (0.11%–0.12%).

### 3.2. Class Comparison Analysis

Class comparison with a significance threshold of *p* < 0.001 was performed after quantile normalization, DWD, and ComBat adjustment of the data and significant antigens were compared between these methods. The overlap between all analyses for investigation of the used normalization and batch removal methods is summarized in Table 3 (details are given in the Supplementary Figure S4). The highest number of significant antigens is achieved upon ComBat and quantile normalization. Much lower numbers are significant when analyzing DWD-adjusted and unnormalized data.

**Table 3:** Number of significant antigens (*p* < 0.001) resulting from class comparison analysis. Rows show results of different data pre-processing methods, columns indicate the analyzed sample group (“r” means experimental run).

Data pre-processing	All (r1-6)	run-wise class comparison				cross-run class comparison			
		SCLC r1	SqLC r2	LCLC r3	AdCa r4	SCLC r1+5	SqLC r2+5	LCLC r3+6	AdCa r4+6
Unnormalized	13	32	17	141	24	74	5	148	5
QNORM	181	340	217	434	98	273	61	362	64
ComBat	299	392	229	500	115	437	109	503	109
DWD	39	34	20	141	35	82	3	176	17

Each histological sample group was processed in a distinct run comprising only samples of one subtype as well as in a mixed run comprising samples of two subtypes and controls. Therefore, class comparison analysis could be performed either run-wise on each histological group, including samples derived from a single run or cross-run analysis was done, including samples of the single run combined with the samples of the respective subtype derived from the mixed run. For each histological subtype, overlaps of the significant antigens derived from class comparison analysis including either data of a single run or data from two combined runs were calculated (Table 4). This enables to investigate if the significant antigens derived from the analysis of a single run remain significant in cross-run analysis. For example, the case of the analysis of the SCLC group, class comparison analysis was done in two ways: including only samples of “run 1”, and combining the samples of “run 1” with the SCLC samples of “run 5”. Then, the overlap of the significant antigens was calculated. This was done using data from each histological group, using all three normalization methods as well as unnormalized data. When comparing the three different normalization strategies, ComBat adjustment yields the highest overlap of significant antigens when comparing analyses from single runs to cross-run analyses, ranging from 25.7% for SqLC to 43.4% for SCLC. With quantile normalization the second highest overlaps have been achieved, ranging from 18.3% for SqLC to 41.6% for SCLC. The lowest number of overlaps was achieved with DWD, which has even lower overlaps for the SCLC (13.7%) and SqLC (9.5%) compared to the unnormalized data (17.8% and 10.0%, respectively). Interestingly, for the histological group of LCLC the normalization method did not influence the overlap at all, ranging from 38.9% to 39.6%. The overlap for AdCa is comparable for all three adjustment methods (30.0% for DWD, 31.7% for quantile normalization, and 38.3% for ComBat).

**Table 4:** Overlap (%) of significant antigens between single-run and cross-run class comparison analysis (*p* < 0.001). This calculation was done with the same data set in an unnormalized state, quantile-normalized, DWD-adjusted data, and ComBat-adjusted data. SCLC = small cell lung cancer, SqLC = squamous cell lung cancer, LCLC = large cell lung cancer, AdCa = adenocarcinoma.

Data pre-processing	class comparison overlap (%) from single-run and cross-run analysis			
	SCLC	SqLC	LCLC	AdCa
unnormalized	17.8	10.0	38.9	7.4
QNORM	41.6	18.3	39.2	31.7
ComBat	43.4	25.7	39.1	38.3
DWD	13.7	9.5	39.6	30.0

**Figure 5:**
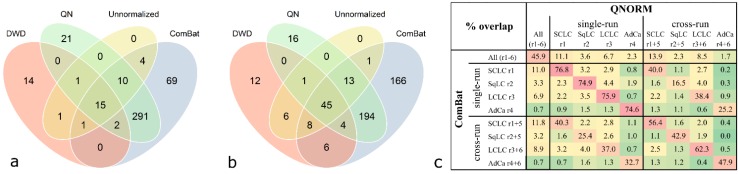
Overlap of significant antigens derived from class comparison analysis (*p* < 0.001) with different normalization methods for the SCLC group. Data was analyzed unnormalized, quantile normalized (QN), DWD-adjusted, and ComBat-adjusted. Significant antigens of SCLC *versus* controls from (**a**) single run analysis (“run-wise” analysis) and (**b**) analysis of two combined experimental runs (“cross-run” analysis). Highest numbers of overlapping antigens are observed between QN and ComBat (318 and 256, respectively). (**c**) Excerpt of Supplementary Figure S4 showing relative overlaps (% representing the intersections of findings depicted in Figure 5a,b) of all class comparison analyses results between quantile normalization and ComBat adjustment.

Comparison of congruent significant antigens derived from class comparison analysis (*p* < 0.001) with different normalization methods revealed that a high proportion of antigens are shared between quantile normalization and ComBat adjustment. This can be observed when analyzing cases and controls derived from one run (exemplified for SCLC in Figure 5a) compared to two processing runs (exemplified for SCLC in Figure 5b). For SCLC, all antigens which are significant in the unnormalized data occur in at least one list of significant antigens of a pre-processed data set. The number of significant antigens in the unnormalized and DWD-adjusted data is very low compared to the quantile-normalized and ComBat-adjusted data. However, a relatively high number of antigens is shared by all four differently pre-processed data sets; 15 antigens in the case of the run-wise analysis and 45 antigens in the case of the cross-run analysis (Figure 5a,b). This can also be observed for the LCLC group, while in the SqLC and the AdCa group the number of overlaps between all four methods is relatively low (see Supplementary Figure S5). 

Comparing relative intersections, the overlaps of significant antigens between quantile normalization and ComBat adjustment ranges from 74.9% to 76.8% for single-run analyses and 42.9% to 62.3% for cross-run analyses (Figure 5c). Considering the overlaps between all adjustment methods as depicted in Supplementary Figure S4, quantile normalization and ComBat adjustment yield the highest values.

The sample size plug-in from BRB-ArrayTools was used to calculate the number of samples needed per sample class for identifying antigens that are differentially reactive between controls and histological subtypes. For this calculation, we selected an accepted type 1 error rate (α) of 0.001, a power (1-β) of 0.9 and a mean difference in log2 expression between classes of 0.585 (equates a fold-change of 1.5). An estimated required sample size of 21, 21, 21, and 20 for SCLC, SqLC, LCLC, and AdCa, respectively, was calculated when using the 50th percentile of the variance distribution. Therefore a good statistical power is given when using 25 samples per group.

### 3.3. Class Prediction Analysis

Class prediction analysis was used to construct multivariate predictors to classify arrays into pre-defined classes based on autoantibody profiles among these classes. The emphasis is to develop an accurate multivariate classifier, which is capable of predicting to which class a future sample belongs. Table 5 provides an overview of class prediction results performed using either the whole data set or only samples having the same histology and matched controls. Although DWD showed the lowest performance in class comparison analysis, this pre-processing method was also included for further class prediction besides quantile normalization and ComBat for evaluation of class prediction performance of all methods. Class prediction analysis of DWD-adjusted data resulted in lower accuracy for the distinct histological entities (79% to 92%) of lung cancer, except for AdCa (91%), compared to quantile normalized and ComBat-adjusted data. In the following, class prediction results of quantile normalized and ComBat-adjusted data are outlined. Different feature selection algorithms and best predictor models have been used to compare the performance of different settings. It can be seen that overall sensitivity and specificity values are higher than 80%. Correct classifications rates, representing the correctly classified samples as case or control using the calculated classifier panel are also comparable, ranging from 79% to 85% when applying class prediction on all lung cancer cases *versus* all controls. Using distinct histological subtypes *versus* matched controls, correct classification rates from 83% to 98% could be achieved. In general, class prediction performed only with data from one histological subtype yielded higher accuracy (0.83–0.98), sensitivity (0.80–1.00) and specificity (0.83–0.96) values than analyzing all lung cancer samples *versus* all controls with an accuracy of 79%–85%, sensitivity of 0.76–0.90, and specificity of 0.75–0.85. Furthermore, it can be seen that when performing class prediction analysis for SCLC or AdCa the quantile normalized data yielded a higher accuracy than ComBat data, whereas for LCLC and SqLC ComBat was favorable. 

**Figure 6:**
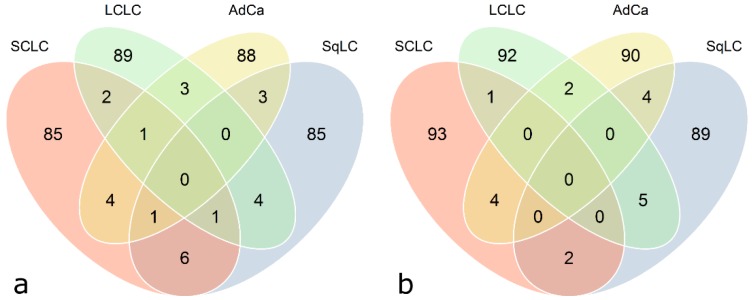
Venn diagram showing classifier overlaps from class prediction results with respect to histological subtypes. Each circle represents a classifier (100 antigens), specific for each histological subtype, found by means of class prediction analysis using recursive feature elimination (see Table 5) as feature selection method. (**a**) Quantile-normalized data, (**b**) ComBat-adjusted data.

Venn diagrams illustrated in Figure 6 represent cross-sectional antigen overlaps calculated between the four histological subtype-specific classifier lists (calculated with class prediction analysis using 100 RFE, Figure 6). Only a minor proportion (zero to six) of antigens does overlap. In general, there are no common antigens among all four histological subtypes. There is almost no difference in the number of overlapping antigenic proteins based on quantile normalized and ComBat adjusted data.

**Table 5:** Performance of classifier panels calculated with class prediction analysis. Different normalization strategies were used and different subsets of samples were analyzed. The analysis of all samples together means that all four histological types of lung cancer were merged to one cancer class.

Analyzed sample set	Data normalization	Feature selection^a^	Classifier size	Best predictor^b^	CC^c^	Sensitivity	Specificity
All cases *vs.* controls (run 1–4)	QN	50 GP	100	1-NN, SVM	83%	0.90	0.75
All cases *vs.* controls (run 1–6)	QN	100 RFE	100	3-NN	79%	0.76	0.83
All cases *vs.* controls (run 1–4)	ComBat	25 GP	50	3-NN	81%	0.85	0.78
All cases *vs.* controls (run 1–6)	ComBat	25 GP	50	SVM	85%	0.85	0.85
SCLC *vs.* control group (run 1 + 5)	QN	100 RFE	100	SVM	98%	1.00	0.96
	ComBat			SVM	94%	0.92	0.96
	DWD			SVM	92%	0.88	0.96
SqLC *vs.* control group (run 2 + 5)	QN	100 RFE	100	SVM	88%	0.80	0.96
	ComBat			CCP, NC, SVM	96%	0.96	0.96
	DWD			SVM	81%	0.76	0.87
LCLC *vs.* control group (run 3 + 6)	QN	100 RFE	100	CCP, SVM	85%	0.84	0.87
	ComBat			1-NN	92%	0.88	0.96
	DWD			SVM	79%	0.72	0.87
AdCa *vs.* control group (run 4 + 6)	QN	100 RFE	100	SVM	89%	0.92	0.87
	ComBat			CCP, DLDA, NC	83%	0.83	0.83
	DWD			DLDA	91%	0.92	0.91

^a^ greedy-pairs algorithm (GP), recursive feature elimination (RFE); ^b^ Nearest Neighbor classification (NN), support vector machine(SVM), Compound Covariate Predictor (CCP), Nearest Centroid (NC), Diagonal Linear Discriminant Analysis (DLDA); ^c^ correct classification rate.

Moreover, the intersection of histological subtype-specific classifiers calculated with class prediction analysis using 100 RFE between ComBat adjusted data and quantile normalized data is approximately 50%. The 100-antigen-classifiers specified by quantile normalization *versus* ComBat for SCLC, SqLC, LCLC, and AdCa were overlapping by 56, 45, 46, and 52 antigens, respectively. The intersection of classifying antigens for DWD *versus* quantile normalization accounts for 13, 15, 18, and 16 antigens, and *versus* ComBat adjustment for 12, 12, 15, and 12 antigens, for SCLC, SqLC, LCLC, and AdCa, respectively.

Classifiers are published and covered in the European Patent Application “Lung cancer diagnostic method and means”, Publication Number EP2806274A1 [43]. Two representative classifiers distinguishing between all cancer cases and cancer-free controls are given in the supplementary section (Supplementary Tables S1 and S2). 

## 4. Discussion 

In the present study, we aimed at comparing normalization and adjustment of data derived from 100 lung cancer cases *versus* 100 matched lung cancer-free controls in terms of effectiveness and influence on subsequent data analysis. Furthermore, the study was focusing on the identification of immune-profiles by means of protein microarrays for early diagnosis of lung cancer. 

Over the past decade, an increasing number of scientific reports have described the presence of autoantibodies, formed in response to TAAs, secreted from solid tumors in sera of cancer patients. In contrast to the present study, other TAA studies [16,53,54,55] use only small panels of already known antigens to determine the extent and frequency of corresponding autoantibodies in cancer samples. We were using protein microarray technology, which allows screening of differentially reactive antigens to detect lung cancer-specific biomarkers. Most recent publications have demonstrated that biomarker research has shifted from single maker analysis to analysis of biomarker signatures using highly parallel methods like microarrays [56]. The number of biomarkers needed to be included in such a panel to establish an efficient and accurate diagnostic tool may vary with the complexity of a disease. Lung cancer, comprising four different main histological entities, is known to be very complex and heterogeneous in terms of its molecular basis [57]. Therefore, it can be expected that biomarker panels rather than a single biomarker would enable lung cancer diagnosis. As the focus of the present study was to compare different data pre-processing strategies we have stated classifiers including 100 antigens to keep the results comparable. Furthermore, the classifiers reported in the present publication are resulting from an initial biomarker screening and will be further validated using a targeted microarray approach. Additionally, higher numbers of candidate antigens were taken into account because these numbers will be reduced when conducting further planned technical platform change to microsphere-based Luminex^®^ system.

We have been using purified IgG samples isolated from plasma instead of diluted serum or plasma, widely used in published studies [9,18,58,59,60,61,62,63,64,65]. IgGs are known to be very stable in serum samples, allowing to detect antibody signatures long after sample taking [66].

The used study population consisted of a well-matched set of lung cancer cases and lung cancer-free controls. To cover the whole histological variety of lung cancer, 25 samples from each of the four main histological subtypes of lung cancer were included in the sample set. Cases and controls were statistically matched for age, sex, and smoking habits to reduce the influence of non-disease-associated effects on the data set. Using 25 samples of each histological subtype of this candidate biomarker discovery, a statistical power of 0.9 could be achieved (and α = 0.001). This emphasizes that the use of 25 biological replicates is sufficient to calculate classifiers with high relevance.

Moreover, the first four runs were designed to include only samples from one distinct histological subtype and matched controls and the last two runs included samples from different histologic types. Certain batch effects or non-biological variations are unavoidable in data sets from large experiments. Especially when involving clinical samples, elaborate study design can decrease the possibility of misleading results. As Luo and colleagues [33] already reported, it is important to carefully remove batch effects from data before starting downstream analysis. Batch effects which are strong enough to mask true biological variations can be removed using data adjustment methods. If the biological issue of the study is completely confounded with the experimental batch design, batch removal methods may also remove true biological variation. In order to avoid that, we considered for our experimental design that in each run cases and controls are intermixed, that the number of cases and controls is the same, and that the maximum possible number of samples belonging to a distinct histological entity is processed in one run. By means of this design, class prediction analyses can be performed on single histological types of lung cancer and may also be performed on the combined data set, including all four entities in order to identify autoantibody-based biomarkers for lung cancer in general. Nevertheless, batch effects cannot be completely avoided though careful design of the study and have to be removed from microarray data sets, especially when several experimental runs have to be conducted. 

Many studies investigate batch effects and removal methods for microarray experiments [28,33,67,68,69]. The first step is to identify batch effects by visualization with PCA [67]. Quantitative evaluation of sources for batch effects can be done by means of PVCA [68]. After investigation of the data with these tools, methods for batch effect removal have to be used before further statistical analysis is performed. Investigation of our microarray data by means of PCA plots revealed that two main groups of samples can be deduced from the unnormalized data. Each group contains three distinct runs of all six experimental runs. One group comprised those runs which were processed chronologically first and the other group included the latter three runs. The difference between those two groups could be due to different days of processing, different stocks of used reagents, or to some other daily circumstances, which are not traceable. Therefore, it is very important to properly record all experiments and to use methods for batch effect removal when combining data sets for further analysis [41]. PVCA analysis conducted on the entire data set showed that the biggest contribution of the batch effect is due to the experimental run. This effect could be removed by the use of DWD and ComBat. Quantile normalization did even result in an increase of the batch effect. Normalization methods are not generally developed for the aim of batch effect removal [33]. It is known that in some cases of microarray experiments batch effects become higher after normalization because the underlying assumptions of normalization methods are transgressed by batch effects [67]. The sample type (case or control) in association with the run showed the second or third strongest influence on variance of the data set, which is most likely due to the biological differences between these groups. Other factors, which were investigated by means of PVCA, were age, sex, and smoking habit of the 200 individuals. None of those variables did contribute to variance in the data. Our sample cohort was statistically matched for these variables between the cancer and the control group in order to exclude any influence of these factors on the analysis results when comparing cancer cases and controls. 

Subsequent to PCA and PVCA analysis class comparison was used in order to retrieve significant (*p* < 0.001) antigens for different sample sets treated with different normalization and data adjustment methods. Overlaps of run-wise and cross-run-derived significant antigen panels were compared for each histological entity of lung cancer (Table 4). Performance of data adjustment methods was evaluated according to the percentage of retained significant antigens when comparing the single run with the cross-run analysis. The lowest number of significant antigens which could be retained when combining two experimental runs in contrast to single-run analysis was observed in unnormalized and DWD-adjusted data. The highest number of retained significant antigens was observed in ComBat-adjusted data, followed by quantile normalization. In respect thereof, ComBat performed the best followed by quantile normalization, although this method did not remove the batch effects successfully. DWD did perform less effective for our data set. Regarding the total number of significant antigens derived from class comparison (Table 3), ComBat outperformed all other methods. The second highest number of significant antigens was found in the quantile normalized data. DWD adjustment did yield low numbers of significant antigens, similar to the unnormalized data, indicating that despite batch effect removal the biological differences might remain masked.

In line with our findings, also other studies found ComBat to perform better than other pre-processing methods [28,41]. Chen *et al.* found ComBat to be superior compared to other batch effect adjustment methods such as DWD and four other methods when using gene expression microarray data. ComBat enabled the reduction of batch effects and increased accuracy and AUC rates [28]. DWD has some limitations of applicability since only two batches can be adjusted at one time. When adjusting more than two batches, this has to be done in a stepwise approach [32]. In the case of our microarray data set, we merged the closest batches according to the PCA plot at first, and the last merge was done between the two clearly separated groups of samples which are shown in the PCA plot of the unnormalized data. This stepwise sequential DWD application could be the potential cause of the low performance of DWD in our protein microarray setting. As presented in Table 4, DWD provided poor results compared to quantile normalization and ComBat. Moreover, the use of DWD as batch adjustment method requires at least 25 samples per batch [31]. Although in our case this requirement could be fulfilled, it could be a problem for other studies with smaller batches.

Since with ComBat and quantile normalization the highest number of significant antigens could be retained when combining experimental runs, further class prediction analysis for identification of tumor autoantibody-based biomarkers for different lung cancer entities and lung cancer in general was done with these two methods. However, to evaluate performance of DWD-adjusted data in class prediction, these analyses were also performed but resulting classifier panels were not further considered because DWD adjustment was less preferred. Comparing accuracy between different data transformation methods showed that DWD adjustment leads to lower correct classification rates compared to quantile normalization and ComBat adjustment, except for the AdCa entity. This may be due to the fact that on the PCA plot of the unnormalized data the experimental run comprising only AdCa and control samples was close to the mixed run which included the remaining AdCa samples. Therefore, these two runs were merged early in the stepwise DWD adjustment. Class prediction analysis was used to calculate classifier panels specific for each histological subtype, which is of great interest for clinical diagnostics. Due to the remarkable molecular heterogeneity of lung cancer, predicting whether a future sample is malignant, combined with classification of tumor histology would be an improvement in targeted cancer therapy [3,70,71,72,73,74]. Already used commercial test kits like the EarlyCDT^®^-Lung assay (Oncimmune^®^ Limited, Nottingham, UK) outperform classical diagnostic methods. By measuring the sero-reactivity against a panel of seven tumor-related antigens (p53, NY-ESO-1, CAGE, GBU4-5, HuD, and MAGE A4, and SOX-2), this assay yields sensitivity and specificity of 41% and 87%, respectively [75,76,77]. However, there is a great need for improving the sensitivity of such assays to cover the whole molecular complexity of lung cancer. This could be achieved by combining already established assays like the EarlyCDT^®^-Lung assay with our identified immuno-profiles which yielded high sensitivity rates since we achieved correct classification of 85% (sensitivity 0.85, specificity 0.85) when using class prediction for the analysis of all lung cancer cases *versus* controls. In this study we were able to calculate histological subtype-specific classifier lists which are only slightly overlapping, meaning that there are only few repeatedly occurring antigens in classifiers of the four histologies. This is in accordance with the well-known molecular heterogeneity of lung cancer and further indicates that future molecular diagnostics of lung cancer need to include specific biomarkers for each histological subtype to provide an accurate and minimal invasive method to initiate targeted and subtype-specific therapy as fast as possible.

## 5. Conclusions 

Since conventional diagnostic methods often fail to detect lung cancer at an early treatable stage, tumor-specific autoantibodies, formed already very early in tumor development, represent an attractive alternative for lung cancer diagnosis. 

In the present study, a protein microarray-based screening for identification of tumor autoantibody markers for lung cancer was performed. In order to evaluate a valid data pre-processing strategy to elucidate reliable candidate classifiers, different normalization and data adjustment methods have been compared. PCA and PVCA plots have shown that only DWD and ComBat, both batch effect removal methods, were able to eliminate non-biological variations from the data set. Further, class comparison analysis yielded the highest number of retained significant antigens when comparing single-run *versus* cross-run analysis of the data sets adjusted with quantile normalization and ComBat. But, as quantile normalization fails to remove batch effects in the data set, we concluded that using ComBat-adjusted data is the best way to pre-process data sets from protein microarrays derived from multiple runs. Class prediction analysis subsequent to ComBat adjustment yielded correct classification rates of 85% for all lung cancer entities combined and even higher correct classification rates for distinct histological lung cancer types ranging from 85% to 98% (Table 5). To the best of our knowledge, these high numbers of correct classification outperform accuracy values of current diagnostic methods.

Handling a large data set and applying different data manipulation algorithms have again shown that adjustment methods and data normalization indeed have a strong influence on subsequent analysis results and should therefore never be applied without critical evaluation using different statistical methods or data visualization tools. Furthermore, with the results of this study we could show that performing a protein microarray-based screening together with an elaborate experimental design, including samples from each main histological subtype of lung cancer, allows detecting tumor-specific autoantibody signatures which are able to cover the whole molecular complexity of lung cancer. However, to fulfill the urgent clinical demand of biomarkers for early diagnosis of lung cancer we encourage the validation of the resulting classifier antigens in larger study populations. Therefore, validation of candidate markers on targeted arrays using planar microarrays or the Luminex^®^ microsphere-based system will be the next step. The data-handling and evaluation procedure as described here would be helpful for biomarker stratification of other cancerous or complex diseases, especially when disease-specific autoantibody signatures or immune-profiles shall be used for biomarker development. Furthermore, for warranting appropriate sample-numbers and statistical power in microarray analyses, multiple experimental runs might have to be conducted in almost all studies. Therefore, the sequential approach to evaluate batch effect removal efficacy and testing the intersection of significant features from single and multiple experimental runs for distinguishing e.g., cases *versus* controls, would be a prerequisite to choose the most reliable candidates from microarrays and other highly multiplexed methods for confirmation and further biomarker development.

## Supplementary Files

Supplementary File 1

## References

[1] Ferlay J., Soerjomataram I., Ervik M., Dikshit R., Eser S., Mathers C., Rebelo M., Parkin D.M., Forman D., Bray F. (2013). GLOBOCAN 2012 v1.0, Cancer Incidence and Mortality Worldwide: IARC CancerBase. No. 11.

[2] Devesa S.S., Bray F., Vizcaino A.P., Parkin D.M. (2005). International lung cancer trends by histologic type: male:female differences diminishing and adenocarcinoma rates rising. Int. J. Cancer.

[3] Petersen I. (2011). The morphological and molecular diagnosis of lung cancer. Dtsch. Arztebl. Int..

[4] Ahn J., Cho J. (2013). Current Serum Lung Cancer Biomarkers. J. Mol. Diagnostics.

[5] Church T.R., Black W.C., Aberle D.R., Berg C.D., Clingan K.L., Duan F., Fagerstrom R.M., Gareen I.F., Gierada D.S., Jones G.C. (2013). Results of initial low-dose computed tomographic screening for lung cancer. N. Engl. J. Med..

[6] Alberg A.J., Brock M.V., Ford J.G., Samet J.M., Spivack S.D. (2013). Epidemiology of lung cancer: Diagnosis and management of lung cancer, 3rd ed: American College of Chest Physicians evidence-based clinical practice guidelines. Chest.

[7] Tan H.T., Low J., Lim S.G., Chung M.C.M. (2009). Serum autoantibodies as biomarkers for early cancer detection. FEBS J..

[8] Anderson K.S., LaBaer J. (2005). The sentinel within: Exploiting the immune system for cancer biomarkers. J. Proteome Res..

[9] Anderson K.S., Sibani S., Wallstrom G., Qiu J., Mendoza E.A., Raphael J., Hainsworth E., Montor W.R., Wong J., Park J.G. (2011). Protein microarray signature of autoantibody biomarkers for the early detection of breast cancer. J. Proteome Res..

[10] Caron M., Choquet-Kastylevsky G., Joubert-Caron R. (2007). Cancer immunomics using autoantibody signatures for biomarker discovery. Mol. Cell. Proteomics.

[11] Casiano C.A., Mediavilla-Varela M., Tan E.M. (2006). Tumor-associated antigen arrays for the serological diagnosis of cancer. Mol. Cell. Proteomics.

[12] Tan E.M., Zhang J. (2008). Autoantibodies to tumor-associated antigens: reporters from the immune system. Immunol. Rev..

[13] Chen G., Wang X., Yu J., Varambally S., Yu J., Thomas D.G., Lin M.-Y., Vishnu P., Wang Z., Wang R. (2007). Autoantibody profiles reveal ubiquilin 1 as a humoral immune response target in lung adenocarcinoma. Cancer Res..

[14] Wang X., Yu J., Sreekumar A., Varambally S., Shen R., Giacherio D., Mehra R., Montie J.E., Pienta K.J., Sanda M.G. (2005). Autoantibody signatures in prostate cancer. N. Engl. J. Med..

[15] Karabudak A.A., Hafner J., Shetty V., Chen S., Secord A.A., Morse M.A., Philip R. (2013). Autoantibody biomarkers identified by proteomics methods distinguish ovarian cancer from non-ovarian cancer with various CA-125 levels. J. Cancer Res. Clin. Oncol..

[16] Chapman C.J., Murray A., McElveen J.E., Sahin U., Luxemburger U., Türeci O., Wiewrodt R., Barnes A.C., Robertson J.F. (2008). Autoantibodies in lung cancer: Possibilities for early detection and subsequent cure. Thorax.

[17] Kazarian M., Laird-Offringa I.A. (2011). Small-cell lung cancer-associated autoantibodies: Potential applications to cancer diagnosis, early detection, and therapy. Mol. Cancer.

[18] Qiu J., Madoz-Gurpide J., Misek D.E., Kuick R., Brenner D.E., Michailidis G., Haab B.B., Omenn G.S., Hanash S. (2004). Development of Natural Protein Microarrays for Diagnosing Cancer Based on an Antibody Response to Tumor Antigens. J. Proteome Res..

[19] Qiu J., Hanash S. (2009). Autoantibody profiling for cancer detection. Clin. Lab. Med..

[20] Ludwig N., Keller A., Leidinger P., Harz C., Backes C., Lenhof H.P., Meese E. (2012). Is there a general autoantibody signature for cancer?. Eur. J. Cancer.

[21] Soussi T. (2000). p53 Antibodies in the sera of patients with various types of cancer: A review. Cancer Res..

[22] Stempfer R., Syed P., Vierlinger K., Pichler R., Meese E., Leidinger P., Ludwig N., Kriegner A., Nöhammer C., Weinhäusel A. (2010). Tumour auto-antibody screening: Performance of protein microarrays using SEREX derived antigens. BMC Cancer.

[23] Kijanka G., Murphy D. (2009). Protein arrays as tools for serum autoantibody marker discovery in cancer. J. Proteomics.

[24] Yang Z., Chevolot Y., Géhin T., Solassol J., Mange A., Souteyrand E., Laurenceau E. (2013). Improvement of protein immobilization for the elaboration of tumor-associated antigen microarrays: Application to the sensitive and specific detection of tumor markers from breast cancer sera. Biosens. Bioelectron..

[25] Lu H., Goodell V., Disis M.L. (2008). Humoral immunity directed against tumor-associated antigens as potential biomarkers for the early diagnosis of cancer. J. Proteome Res..

[26] Mou Z., He Y., Wu Y. (2009). Immunoproteomics to identify tumor-associated antigens eliciting humoral response. Cancer Lett..

[27] Quackenbush J. (2002). Microarray data normalization and transformation. Nat. Genet..

[28] Chen C., Grennan K., Badner J., Zhang D., Gershon E., Jin L., Liu C. (2011). Removing batch effects in analysis of expression microarray data: an evaluation of six batch adjustment methods. PLoS One.

[29] Yang Y.H., Buckley M.J., Dudoit S., Speed T.P. (2002). Comparison of Methods for Image Analysis on cDNA Microarray Data. J. Comput. Graph. Stat..

[30] Bolstad B.M., Irizarry R.A., Astrand M., Speed T.P. (2003). A comparison of normalization methods for high density oligonucleotide array data based on variance and bias. Bioinformatics.

[31] Johnson W.E., Li C., Rabinovic A. (2007). Adjusting batch effects in microarray expression data using empirical Bayes methods. Biostatistics.

[32] Benito M., Parker J., Du Q., Wu J., Xiang D., Perou C.M., Marron J.S. (2004). Adjustment of systematic microarray data biases. Bioinformatics.

[33] Luo J., Schumacher M., Scherer A., Sanoudou D., Megherbi D., Davison T., Shi T., Tong W., Shi L., Hong H. (2010). A comparison of batch effect removal methods for enhancement of prediction performance using MAQC-II microarray gene expression data. Pharmacogenomics J..

[34] Li J., Bushel P.R., Chu T.-M., Wolfinger R.D., Scherer A. (2009). Principal Variance Components Analysis: Estimating Batch Effects in Microarray Gene Expression Data. Batch Effects and Noise in Microarray Experiments: Sources and Solutions.

[35] Simon R., Lam A., Li M.-C., Ngan M., Menenzes S., Zhao Y. (2007). Analysis of gene expression data using BRB-ArrayTools. Cancer Inform..

[36] Dobbin K., Simon R. (2005). Sample size determination in microarray experiments for class comparison and prognostic classification. Biostatistics.

[37] Zhang D.Y., Ye F., Gao L., Liu X., Zhao X., Che Y., Wang H., Wang L., Wu J., Song D. (2009). Proteomics, pathway array and signaling network-based medicine in cancer. Cell Div..

[38] Simon R.M., Mcshane L.M., Wright G.W., Korn E.L., Radmacher M.D., Zhao Y. (2009). Design and Analysis of DNA Microatray Investigations.

[39] Simon R., Radmacher M.D., Dobbin K., McShane L.M. (2003). Pitfalls in the Use of DNA Microarray Data for Diagnostic and Prognostic Classification. J. Natl. Cancer Inst..

[40] Simon R. (2008). Microarray Based Expression Profiling and Informatics. Curr. Opin. Biotechnol..

[41] Bevilacqua V., Pannarale P., Abbrescia M., Cava C., Paradiso A., Tommasi S. (2012). Comparison of data-merging methods with SVM attribute selection and classification in breast cancer gene expression. BMC Bioinformatics.

[42] Park T., Yi S., Kang S., Lee S., Lee Y.-S., Simon R. (2003). Evaluation of normalization methods for microarray data. BMC Bioinformatics.

[43] Weinhäusel A. Lung cancer diagnostic method and means. European Patent Application.

[44] Collin M., Olsén A. (2001). EndoS, a novel secreted protein from Streptococcus pyogenes with endoglycosidase activity on human IgG. EMBO J..

[45] Pless O., Kowenz-Leutz E., Dittmar G., Leutz A. (2011). A differential proteome screening system for post-translational modification-dependent transcription factor interactions. Nat. Protoc..

[46] Bø T., Jonassen I. (2002). New feature subset selection procedures for classification of expression profiles. Genome Biol..

[47] Hastie T., Tibshirani R., Narasimhan B., Chu G. Impute: Imputation For Microarray Data.

[48] R Core Team (2013). R: A Language and Environment for Statistical Computing.

[49] Bushel P. PVCA: Principal Variance Component Analysis (PVCA).

[50] Guyon I., Weston J., Barnhill S., Vapnik V. (2002). Gene Selection for Cancer Classification using Support Vector Machines. Mach. Learn..

[51] Rosskopf S., Gyurján I., Luna-Coronell J.A., Vierlinger K., Singer C., Weinhäusel A. (2015). The pre-analytical processing of blood samples for detecting biomarkers on protein microarrays. J. Immunol. Methods.

[52] Ringnér M. (2008). What is principal component analysis?. Nat. Biotechnol..

[53] Looi K., Megliorino R., Shi F.-D., Peng X.-X., Chen Y., Zhang J.-Y. (2006). Humoral immune response to p16, a cyclin-dependent kinase inhibitor in human malignancies. Oncol. Rep..

[54] Yagihashi A., Asanuma K., Kobayashi D., Tsuji N., Shijubo Y., Abe S., Hirohashi Y., Torigoe T., Sato N., Watanabe N. (2005). Detection of autoantibodies to livin and survivin in sera from lung cancer patients. Lung Cancer.

[55] Zhang J.-Y., Casiano C.A., Peng X.-X., Koziol J.A., Chan E.K. L., Tan E.M. (2003). Enhancement of antibody detection in cancer using panel of recombinant tumor-associated antigens. Cancer Epidemiol. Biomarkers Prev..

[56] Camps C., Jantus-Lewintre E., Usó M., Sanmartin E. (2012). Update on biomarkers for the detection of lung cancer. Lung Cancer Targets Ther..

[57] Cooper W.A., Lam D.C. L., O’Toole S.A., Minna J.D. (2013). Molecular biology of lung cancer. J. Thorac. Dis..

[58] Fernández-Madrid F., VandeVord P.J., Yang X., Karvonen R.L., Simpson P.M., Kraut M.J., Granda J.L., Tomkiel J.E. (1999). Antinuclear antibodies as potential markers of lung cancer. Clin. Cancer Res..

[59] Nakanishi T., Takeuchi T., Ueda K., Murao H., Shimizu A. (2006). Detection of eight antibodies in cancer patients’ sera against proteins derived from the adenocarcinoma A549 cell line using proteomics-based analysis. J. Chromatogr. B Anal. Technol. Biomed. Life Sci..

[60] Zayakin P., Ancāns G., Siliņa K., Meistere I., Kalniņa Z., Andrejeva D., Endzeliņš E., Ivanova L., Pismennaja A., Ruskule A. (2013). Tumor-associated autoantibody signature for the early detection of gastric cancer. Int. J. Cancer.

[61] Zhang J.Y., Zhu W., Imai H., Kiyosawa K., Chan E.K. L., Tan E.M. (2001). De-novo humoral immune responses to cancer-associated autoantigens during transition from chronic liver disease to hepatocellular carcinoma. Clin. Exp. Immunol..

[62] Zhang J.-Y., Megliorino R., Peng X.-X., Tan E.M., Chen Y., Chan E.K.L. (2007). Antibody detection using tumor-associated antigen mini-array in immunodiagnosing human hepatocellular carcinoma. J. Hepatol..

[63] Lin H.S., Talwar H.S., Tarca A.L., Ionan A., Chatterjee M., Ye B., Wojciechowski J., Mohapatra S., Basson M.D., Yoo G.H. (2007). Autoantibody approach for serum-based detection of head and neck cancer. Cancer Epidemiol. Biomarkers Prev..

[64] Syed P., Gyurján I., Kriegner A., Vierlinger K., Singer C.F., Rappaport-fürhauser C., Zerweck J., Söllner J. (2012). In silico design and performance of peptide microarrays for breast cancer tumour auto-antibody testing. J. Mol. Biochem..

[65] Syed P., Vierlinger K. (2012). Evaluation of auto-antibody serum biomarkers for breast cancer screening and in silico analysis of sero-reactive proteins. J. Mol. Biochem..

[66] Gislefoss R.E., Grimsrud T.K., Mørkrid L. (2009). Stability of selected serum proteins after long-term storage in the Janus Serum Bank. Clin. Chem. Lab. Med..

[67] Leek J.T., Scharpf R.B., Bravo H.C., Simcha D., Langmead B., Johnson W.E., Geman D., Baggerly K., Irizarry R.A. (2010). Tackling the widespread and critical impact of batch effects in high-throughput data. Nat. Rev. Genet..

[68] Lazar C., Meganck S., Taminau J., Steenhoff D., Coletta A., Molter C., Weiss-Solís D.Y., Duque R., Bersini H., Nowé A. (2013). Batch effect removal methods for microarray gene expression data integration: a survey. Brief. Bioinform..

[69] Kitchen R., Sabine V., Sims A. (2010). Correcting for intra-experiment variation in Illumina BeadChip data is necessary to generate robust gene-expression profiles. BMC Genomics.

[70] Borczuk A.C., Gorenstein L., Walter K.L., Assaad A.A., Wang L., Powell C.A. (2003). Non-small-cell lung cancer molecular signatures recapitulate lung developmental pathways. Am. J. Pathol..

[71] Cetin K., Ettinger D.S., Hei Y.-J., O’Malley C.D. (2011). Survival by histologic subtype in stage IV nonsmall cell lung cancer based on data from the Surveillance, Epidemiology and End Results Program. Clin. Epidemiol..

[72] Dacic S. (2011). Molecular diagnostics of lung carcinomas. Arch. Pathol. Lab. Med..

[73] Selvaggi G., Scagliotti G.V. (2009). Histologic subtype in NSCLC: Does it matter?. Oncology.

[74] West L., Vidwans S.J., Campbell N.P., Shrager J., Simon G.R., Bueno R., Dennis P.A., Otterson G.A., Salgia R. (2012). A novel classification of lung cancer into molecular subtypes. PLoS One.

[75] Chapman C.J., Healey G.F., Murray A., Boyle P., Robertson C., Peek L.J., Allen J., Thorpe A.J., Hamilton-Fairley G., Parsy-Kowalska C.B. (2012). EarlyCDT^®^-Lung test: Improved clinical utility through additional autoantibody assays. Tumour Biol..

[76] Lam S., Boyle P., Healey G.F., Maddison P., Peek L., Murray A., Chapman C.J., Allen J., Wood W.C., Sewell H.F. (2011). EarlyCDT-Lung: An immunobiomarker test as an aid to early detection of lung cancer. Cancer Prev. Res. (Phila)..

[77] Jett J.R., Peek L.J., Fredericks L., Jewell W., Pingleton W.W., Robertson J.F.R. (2014). Audit of the autoantibody test, EarlyCDT^®^-Lung, in 1600 patients: An evaluation of its performance in routine clinical practice. Lung Cancer.

