# Supplementary material for: Immune-Signatures for Lung Cancer Diagnostics: Evaluation of Protein Microarray Data Normalization Strategies
Source: Microarrays (Basel). 2015 Apr 2;4(2):162–87. doi: 10.3390/microarrays4020162 (PMC4996396; doi:10.3390/microarrays4020162)
Supplement: Supplementary File 1 [file microarrays-04-00162-s001.pdf]

Supplementary Materials

Immune-Signatures for Lung Cancer Diagnostics: Evaluation of Protein Microarray Data Normalization Strategies

Stefanie Brezina, Regina Soldo, Roman Kreuzhuber, Philipp Hofer, Andrea Gsur and Andreas Weinhaeusel

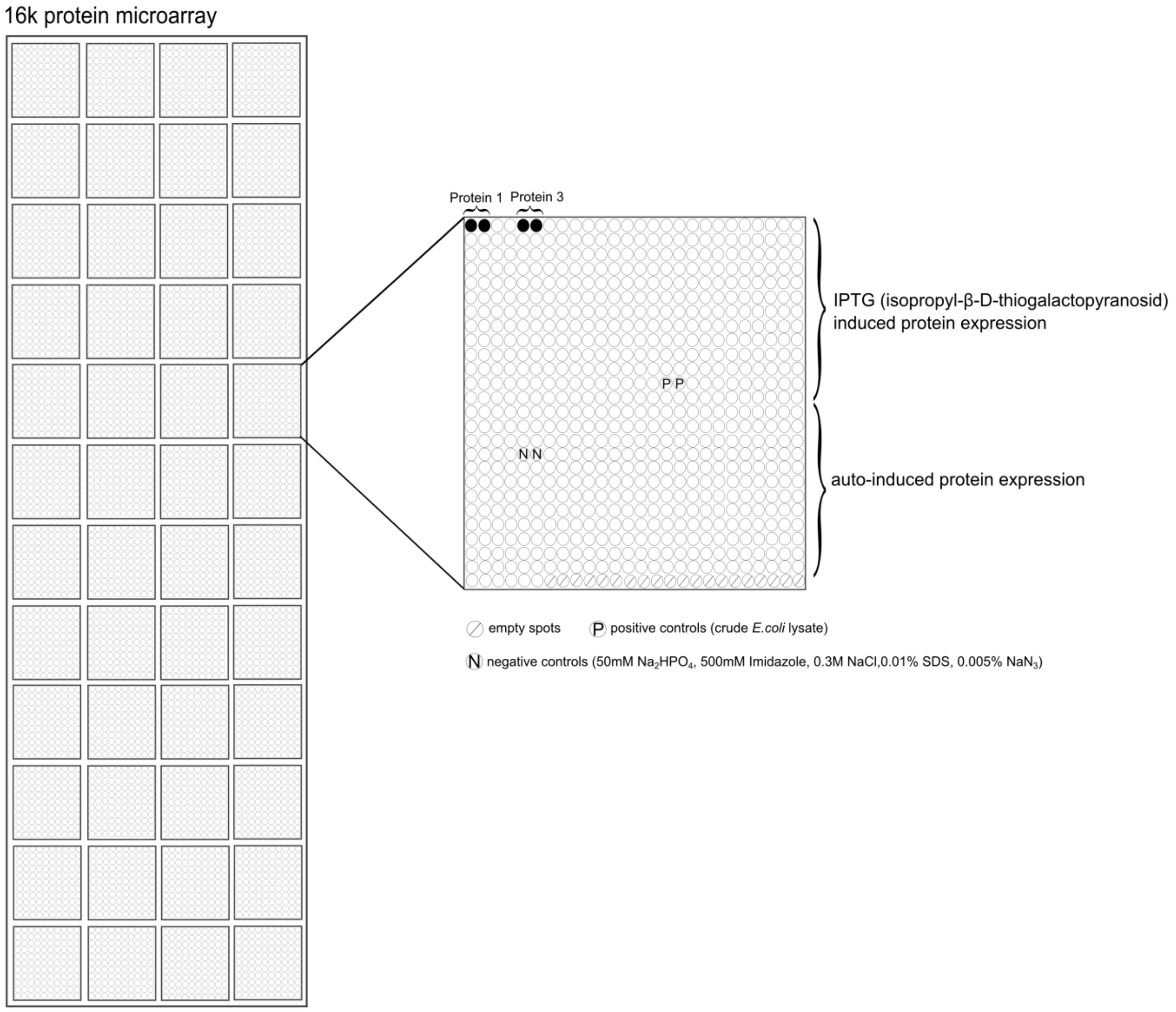

Figure S1. 16k protein microarray design. The array is sub-divided into 48 blocks, each containing 676 spots. Protein spots are printed in duplicate. As positive control crude *E. coli* lysate (0.3, 0.4, and 0.5 mg mL^{-1}) and as negative control elution buffer was spotted onto the slides. Control spots are randomly distributed over the whole array. In total, 281 positive and 82 negative control spots are printed onto the 16k microarray.

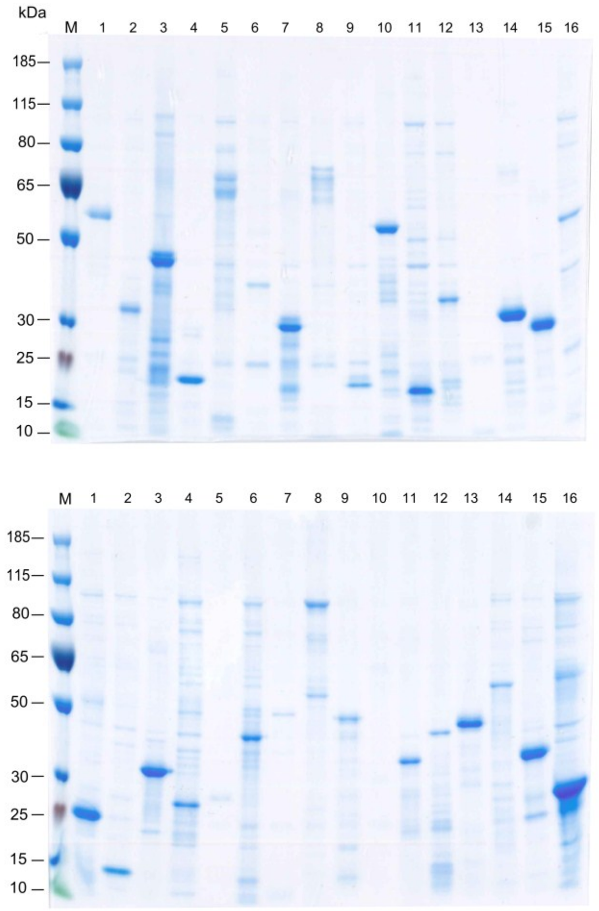

Figure S2. SDS-PAGE (NuPAGE Novex, 4%–12% Bis-tris) of 2 µg UniPEx protein eluates (lanes 1–16) after Ni-NTA purification. PageRuler™ Plus Prestained Protein Ladder (Life Technologies, Carlsbad, CA, USA) was used.

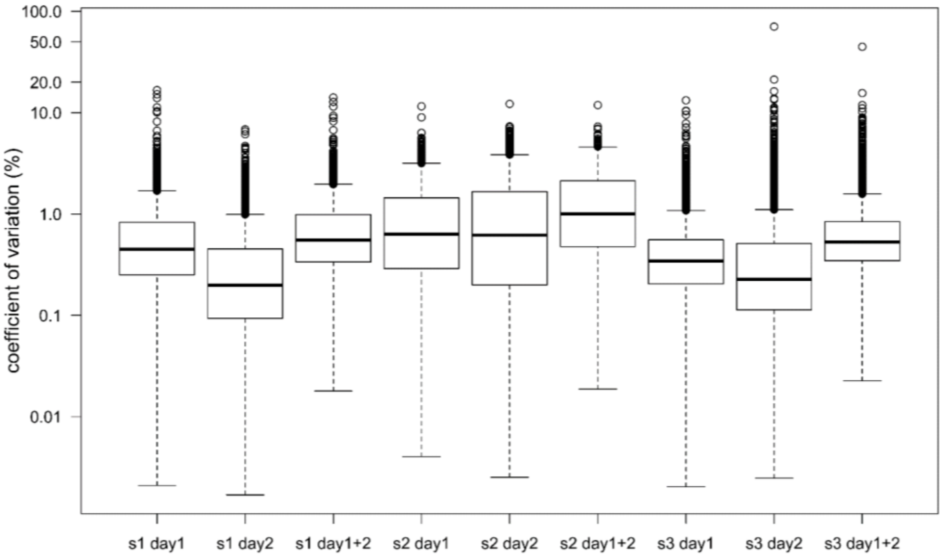

Figure S3. Boxplot representing intra-experiment and inter-experiment coefficient of variation (CV) values of all proteins on the 16k microarray. Each box represents a replicate of a specimen (s1, s2, s3) which was processed on the microarray in triplicates on the same day (day1 and day2 = intra-experiment comparison) and on consecutive days (day1+2= inter-experiment comparison).

% overlap			DWD									ComBat									QNORM									Unnormalized								
				single-run				cross-run					single-run				cross-run					single-run				cross-run					single-run				cross-run			
			All (r1-6)	SCLC r1	SqLC r2	LCLC r3	AdCa r4	SCLC r1+5	SqLC r2+5	LCLC r3+6	AdCa r4+6	All (r1-6)	SCLC r1	SqLC r2	LCLC r3	AdCa r4	SCLC r1+5	SqLC r2+5	LCLC r3+6	AdCa r4+6	All (r1-6)	SCLC r1	SqLC r2	LCLC r3	AdCa r4	SCLC r1+5	SqLC r2+5	LCLC r3+6	AdCa r4+6	All (r1-6)	SCLC r1	SqLC r2	LCLC r3	AdCa r4	SCLC r1+5	SqLC r2+5	LCLC r3+6	AdCa r4+6
DWD		All (r1-6)	39	1.4	0.0	1.7	12.1	0.0	0.0	0.9	16.7	4.6	0.7	2.3	1.9	6.2	1.1	2.8	1.3	5.0	4.8	1.3	1.6	1.9	3.8	1.3	1.0	1.5	2.0	10.6	1.4	0.0	2.3	5.0	0.0	0.0	2.2	0.0
	single-run	SCLC r1	1	34	0.0	0.0	0.0	13.7	0.0	0.0	0.0	1.2	4.4	1.2	0.2	0.0	3.7	0.0	0.2	0.0	1.4	5.1	1.2	0.2	0.0	5.5	0.0	0.5	0.0	9.3	37.5	0.0	0.0	0.0	12.5	0.0	0.0	0.0
		SqLC r2	0	0	20	2.5	1.9	0.0	9.5	1.0	0.0	0.0	0.5	5.5	0.4	1.5	0.0	5.7	0.0	1.6	0.0	0.0	6.8	0.4	0.0	0.0	6.6	0.0	0.0	0.0	0.0	42.3	1.9	0.0	0.0	4.2	0.0	0.0
		LCLC r3	3	0	4	141	0.0	0.0	0.0	39.6	0.0	0.9	0.8	5.4	12.1	1.2	0.7	4.2	7.7	2.0	0.3	1.1	5.3	12.1	0.0	0.5	3.1	9.8	1.5	0.0	0.0	1.3	65.9	0.0	0.0	0.0	29.6	0.7
		AdCa r4	8	0	1	0	35	0.0	0.0	0.0	30.0	0.6	0.0	1.9	0.2	16.3	0.2	2.9	0.0	9.9	0.9	0.3	1.2	0.4	16.7	0.0	1.1	0.3	7.6	2.1	0.0	0.0	0.0	34.1	0.0	0.0	0.0	2.6
	cross-run	SCLC r1+5	0	14	0	0	0	82	0.0	0.8	0.0	5.0	12.6	0.3	0.7	0.0	13.8	0.0	1.0	0.0	4.8	14.1	0.7	1.0	0.6	16.4	0.0	1.1	0.0	2.2	9.6	0.0	0.0	0.0	62.5	0.0	0.4	0.0
		SqLC r2+5	0	0	2	0	0	0	3	0.0	0.0	0.0	0.0	0.4	0.0	0.0	0.0	0.0	0.0	0.0	0.0	0.0	0.5	0.0	0.0	0.0	0.0	0.0	0.0	0.0	0.0	5.3	0.0	0.0	0.0	14.3	0.0	0.0
		LCLC r3+6	2	0	2	90	0	2	0	176	0.0	1.7	1.6	4.4	17.0	0.3	1.3	2.5	17.3	0.7	1.4	1.8	4.5	17.3	0.0	1.6	2.2	20.4	0.8	0.0	0.0	0.0	29.9	0.0	0.8	0.0	48.6	0.0
		AdCa r4+6	8	0	0	0	12	0	0	0	17	0.0	0.0	2.5	0.0	10.0	0.2	4.1	0.0	8.6	0.0	0.3	1.3	0.2	8.5	0.0	1.3	0.3	3.8	0.0	0.0	0.0	0.0	17.1	0.0	0.0	0.0	4.8
ComBat		All (r1-6)	15	4	0	4	2	18	0	8	0	299	10.7	3.1	8.0	2.0	16.3	3.6	11.2	1.5	45.9	11.1	3.6	6.7	2.3	13.9	2.3	8.5	1.7	3.3	1.5	0.0	1.1	0.6	6.3	0.3	1.1	0.3
	single-run	SCLC r1	3	18	2	4	0	53	0	9	0	67	392	2.6	2.3	1.0	43.4	1.4	3.5	0.8	11.0	76.8	3.2	2.9	0.8	40.0	1.1	2.7	0.2	1.0	7.6	1.2	0.8	0.0	14.2	0.0	1.5	0.0
		SqLC r2	6	3	13	19	5	1	1	17	6	16	16	229	3.8	2.4	1.8	25.7	4.0	2.1	3.3	2.3	74.9	4.4	1.9	1.6	16.5	4.0	0.3	0.8	0.8	6.0	5.7	0.8	0.3	1.3	5.0	0.0
		LCLC r3	10	1	2	69	1	4	0	98	0	59	20	27	500	1.0	2.3	2.0	39.1	1.0	6.9	2.2	3.5	75.9	0.7	2.2	1.4	38.4	0.9	0.8	0.0	0.4	13.9	0.0	0.5	0.0	16.5	0.2
		AdCa r4	9	0	2	3	21	0	0	1	12	8	5	8	6	115	0.9	2.3	0.8	38.3	0.7	0.9	1.5	1.3	74.6	1.3	1.1	0.6	25.2	0.8	0.0	0.8	0.8	11.2	0.0	0.0	0.0	0.0
	cross-run	SCLC r1+5	5	17	0	4	1	63	0	8	1	103	251	12	21	5	437	1.7	2.6	1.3	11.8	40.3	2.2	2.8	1.1	56.4	1.6	2.0	0.4	1.1	5.4	0.7	0.9	0.4	15.1	0.0	0.9	0.0
		SqLC r2+5	4	0	7	10	4	0	0	7	5	14	7	69	12	5	9	109	1.5	1.9	3.2	1.6	25.4	2.6	1.0	1.1	42.9	1.9	0.0	0.0	0.0	7.7	4.2	0.8	0.0	2.7	3.2	0.0
		LCLC r3+6	7	1	0	46	0	6	0	100	0	81	30	28	282	5	24	9	503	0.5	8.9	3.2	4.0	37.0	0.7	2.5	1.3	62.3	0.5	0.8	0.0	0.0	9.0	0.0	0.7	0.2	17.1	0.0
		AdCa r4+6	7	0	2	5	13	0	0	2	10	6	4	7	6	62	7	4	3	109	0.7	0.7	1.6	1.3	32.7	1.3	1.2	0.4	47.9	0.8	0.0	0.8	1.2	9.0	0.0	0.0	0.0	1.8
QNORM		All (r1-6)	10	3	0	1	2	12	0	5	0	151	57	13	44	2	65	9	56	2	181	10.4	3.4	6.4	0.7	13.2	3.0	8.0	0.8	4.3	2.4	0.0	1.3	1.5	5.8	0.5	1.5	0.5
	single-run	SCLC r1	5	18	0	5	1	52	0	9	1	64	318	13	18	4	223	7	26	3	49	340	2.8	2.8	0.9	41.6	0.8	2.6	0.2	1.1	7.5	0.3	0.8	0.0	15.6	0.0	1.0	0.0
		SqLC r2	4	3	15	18	3	2	1	17	3	18	19	191	24	5	14	66	28	5	13	15	217	4.2	1.6	2.1	18.3	3.9	0.4	0.9	0.8	6.8	5.6	0.4	0.7	1.4	5.2	0.0
		LCLC r3	9	1	2	62	2	5	0	90	1	46	23	28	403	7	24	14	253	7	37	21	26	434	0.95	3.1	2.1	39.2	1.0	0.7	0.0	0.7	14.3	0.2	0.8	0.0	17.3	0.2
		AdCa r4	5	0	0	0	19	1	0	0	9	9	4	6	4	91	6	2	4	51	2	4	5	5	98	0.8	1.3	0.7	31.7	0.9	0.0	0.0	0.0	14.0	0.6	0.0	0.0	0.0
	cross-run	SCLC r1+5	4	16	0	2	0	50	0	7	0	70	190	8	17	5	256	4	19	5	53	180	10	21	3	273	1.5	2.3	0.6	1.8	7.8	0.7	0.7	0.3	20.5	0.0	0.7	0.0
		SqLC r2+5	1	0	5	6	1	0	0	5	1	8	5	41	8	2	8	51	7	2	7	3	43	10	2	5	61	1.7	0.0	0.0	0.0	9.9	3.6	1.2	0.0	4.8	3.0	0.0
		LCLC r3+6	6	2	0	45	1	5	0	91	1	52	20	23	239	3	16	9	332	2	40	18	22	224	3	14	7	362	0.5	1.1	0.0	0.0	12.0	0.3	0.9	0.0	21.7	0.0
		AdCa r4+6	2	0	0	3	7	0	0	2	3	6	1	1	5	36	2	0	3	56	2	1	1	5	39	2	0	2	64	1.3	0.0	0.0	0.5	10.0	0.0	0.0	0.0	3.0
Unnormalized		All (r1-6)	5	4	0	0	1	2	0	0	0	10	4	2	4	1	5	0	4	1	8	4	2	3	1	5	0	4	1	13	4.7	0.0	0.0	2.8	2.4	0.0	0.0	0.0
	single-run	SCLC r1	1	18	0	0	0	10	0	0	0	5	30	2	0	0	24	0	0	0	5	26	2	0	0	22	0	0	0	2	32	0.0	0.0	0.0	17.8	0.0	0.0	0.0
		SqLC r2	0	0		2	0	0	1	0	0	0	5	14	2	1	3	9	0	1	0	1	15	3	0	2	7	0	0	0	0	17	1.9	0.0	0.0	10.0	0.0	0.0
		LCLC r3	4	0	3	112	0	0	0	73	0	5	4	20	78	2	5	10	53	3	4	4	19	72	0	3	7	54	1	0	0	3	141	0.0	0.0	0.0	38.9	0.7
		AdCa r4	3	0	0	0	15	0	0	0	6	2	0	2	0	14	2	1	0	11	3	0	1	1	15	1	1	1	8	1	0	0	0	24	0.0	0.0	0.0	7.4
	cross-run	SCLC r1+5	0	12	0	0	0	60	0	2	0	22	58	1	3	0	67	0	4	0	14	56	2	4	1	59	0	4	0	2	16	0	0	0	74	0.0	0.5	0.0
		SqLC r2+5	0	0	1	0	0	0	1	0	0	1	0	3	0	0	0	3	1	0	1	0	3	0	0	0	3	0	0	0	0	2	0	0	0	5	0.0	0.0
		LCLC r3+6	4	0	0	66	0	1	0	106	0	5	8	18	92	0	5	8	95	0	5	5	18	86	0	3	6	91	0	0	0	0	81	0	1	0	148	0.0
		AdCa r4+6	0	0	0	1	1	0	0	0	1	1	0	0	1	0	0	0	0	2	1	0	0	1	0	0	0	0	2	0	0	0	1	2	0	0	0	5

% overlap

absolute overlap

Figure S4. Overlaps of significant antigens derived from class comparison analysis with different data pre-processing methods (DWD, ComBat, quantile normalization, unnormalized) including the whole data set with all lung cancer cases *versus* controls ("All (r1-6)"), and "single-run" as well as "cross-run" analyses of the four histologic entities. Right upper half shows relative overlaps (%) and left lower half shows absolute overlaps. QNORM = quantile normalization, SCLC = small cell lung cancer, SqLC = squamous cell lung cancer, LCLC = large cell lung cancer, AdCa = adenocarcinoma of the lung, r = experimental run.

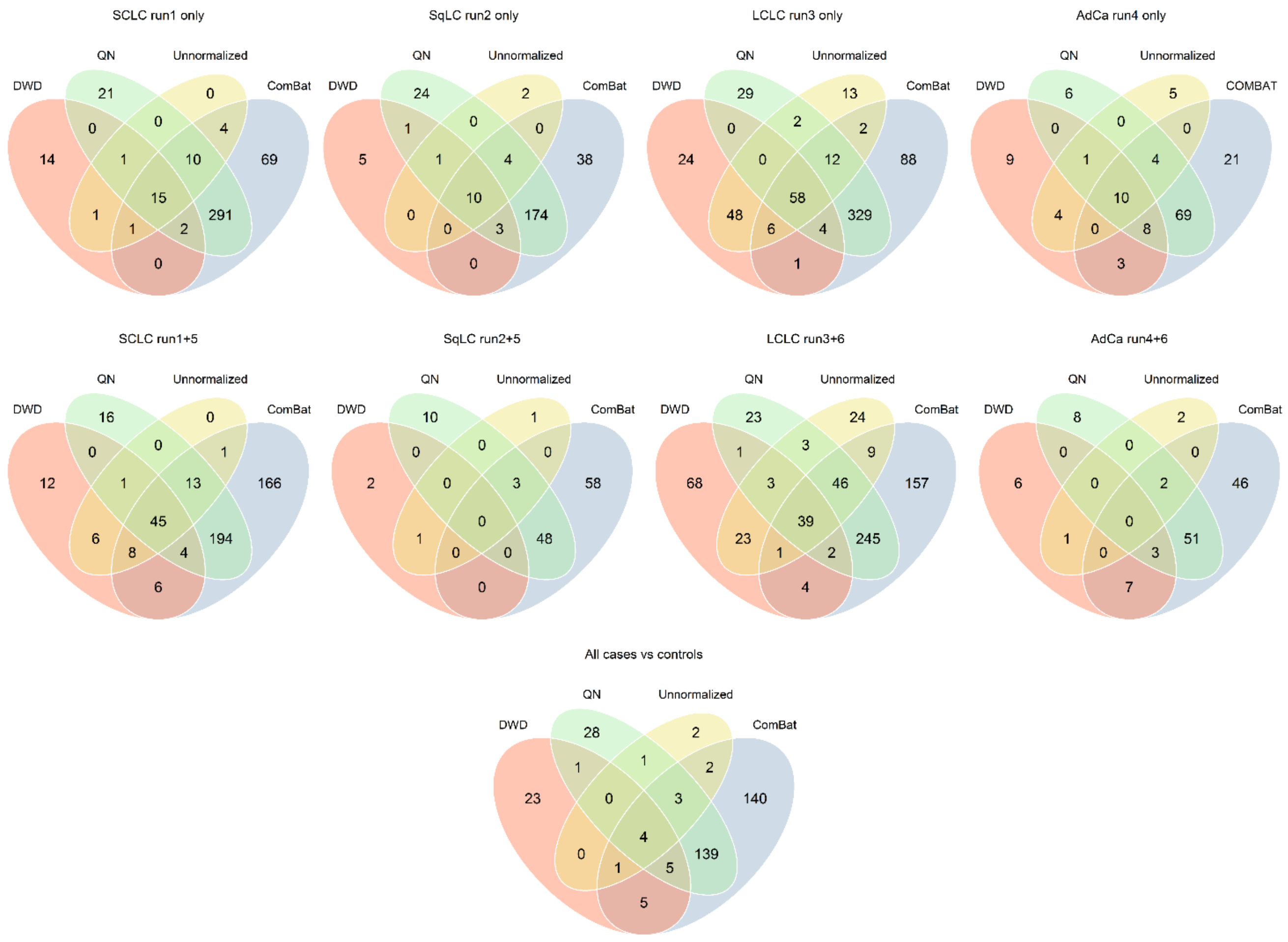

Figure S5. Overlaps of significant antigens derived from class comparison analysis ($p < 0.001$) with different data pre-processing methods (DWD, QN = quantile normalization, unnormalized, ComBat) for “single-run” analysis (run1 only to run4 only) and “cross-run” analysis (run1+5 to run4+6) for distinct histologic entities of lung cancer (SCLC = small cell lung cancer, SqLC = squamous cell lung cancer, LCLC = large cell lung cancer, AdCa = adenocarcinoma of the lung), as well as for all cases *versus* all controls including all samples.

Table S1. Classifier list established with class prediction using quantile normalized data and 100 recursive feature elimination using all cases (n = 100) *versus* all controls (n = 100). For class prediction results compare Table 5.

UniqueID	Gene Symbol	*p*-value	UniqueID	Gene Symbol	*p*-value
RZPDp9027L0419Q	KIF5A	1.00×10^{-6}	RZPDp9027G0320Q	TXN2	1.35×10^{-1}
RZPDp9028L0418Q	EIF3M	1.05×10^{-4}	RZPDp9028M0217Q	TOMM20	1.30×10^{-1}
RZPDp9028L1618Q	NFYA	1.32×10^{-4}	RZPDp9027G197Q	NOL11	1.30×10^{-1}
RZPDp9028L1514Q	FPGS	1.96×10^{-4}	RZPDp9027D1316Q	NECAP1	1.13×10^{-1}
RZPDp9028G0420Q	NFKBIA	2.91×10^{-4}	RZPDp9028C229Q	SNRNP48	9.64×10^{-2}
RZPDp9027G0711Q	TRIOBP	3.61×10^{-4}	RZPDp9028K2012Q	ARHGEF1	9.59×10^{-2}
RZPDp9027H168Q	BCAS2	7.07×10^{-4}	RZPDp9028L201Q	LRP1	6.11×10^{-2}
RZPDp9028O1314Q	HLA-E	1.93×10^{-3}	RZPDp9027E1817Q	EXOSC10	5.74×10^{-2}
RZPDp9028N0516Q	ADI1	2.13×10^{-3}	RZPDp9028B0517Q	PPP6R1	5.62×10^{-2}
RZPDp9028K1819Q	TBCB	2.34×10^{-3}	RZPDp9027I1618Q	GABBR1	5.61×10^{-2}
RZPDp9028J0319Q	FYN	2.92×10^{-3}	RZPDp9028N1413Q	TP53BP2	5.48×10^{-2}
RZPDp9027K1912Q	TRAK1	3.07×10^{-3}	RZPDp9028G059Q	AKAP13	5.06×10^{-2}
RZPDp9028L1519Q	ARHGEF18	4.83×10^{-3}	RZPDp9028I1316Q	EDARADD	4.99×10^{-2}
RZPDp9028A0112Q	TRIM28	9.32×10^{-3}	RZPDp9028H1220Q	RRP1B	4.68×10^{-2}
RZPDp9027K1911Q	SNCB	1.02×10^{-2}	RZPDp9028B0915Q	DUSP2	4.14×10^{-2}
RZPDp9028K169Q	PRMT1	1.05×10^{-2}	RZPDp9028F218Q	ATXN2L	4.08×10^{-2}
RZPDp9027C1912Q	TMEM222	1.35×10^{-2}	RZPDp9028A1219Q	NLRP1	2.27×10^{-2}
RZPDp9028N136Q	MUC2	1.55×10^{-2}	RZPDp9028F019Q	HERC2	2.26×10^{-2}
RZPDp9028K0714Q	NFKB1	2.04×10^{-2}	RZPDp9028P168Q	CEP250	2.17×10^{-2}
RZPDp9027I062Q	G3BP2	2.15×10^{-2}	RZPDp9027G1218Q	EPS8	1.98×10^{-2}
RZPDp9028O1617Q	PLCG1	2.98×10^{-2}	RZPDp9028C2019Q	GGA2	1.92×10^{-2}
RZPDp9028H1517Q	ACO2	3.14×10^{-2}	RZPDp9027A196Q	CUL7	1.77×10^{-2}
RZPDp9028D191Q	COMP	3.66×10^{-2}	RZPDp9028A1014Q	NLRC5	1.64×10^{-2}
RZPDp9028G0616Q	APBB1	3.76×10^{-2}	RZPDp9028D058Q	UTP14A	1.54×10^{-2}
RZPDp9027G0710Q	FAM192A	3.99×10^{-2}	RZPDp9028P024Q	ARHGDIA	1.26×10^{-2}
RZPDp9028P028Q	NEDD9	4.35×10^{-2}	RZPDp9028J074Q	SLC9A3R2	1.14×10^{-2}
RZPDp9028D093Q	COL4A1	6.78×10^{-2}	RZPDp9027D2118Q	COPE	1.04×10^{-2}
RZPDp9028P048Q	ERCC5	7.07×10^{-2}	RZPDp9028I186Q	AGRN	1.01×10^{-2}
RZPDp9027G078Q	C10orf35	7.16×10^{-2}	RZPDp9028G0917Q	SETD2	5.60×10^{-3}
RZPDp9028I0219Q	D2HGDH	8.84×10^{-2}	RZPDp9028L2017Q	RPS25	4.88×10^{-3}
RZPDp9028N152Q	CCDC88C	9.99×10^{-2}	RZPDp9028N0410Q	FBF1	4.74×10^{-3}
RZPDp9027J039Q	SBK1	1.13×10^{-1}	RZPDp9027E197Q	AKR1C4	1.52×10^{-3}
RZPDp9028G1711Q	RSBN1	1.46×10^{-1}	RZPDp9028A0915Q	SMYD5	1.48×10^{-3}
RZPDp9028I021Q	IGHG1	1.52×10^{-1}	RZPDp9028H1210Q	NFKB1	1.29×10^{-3}
RZPDp9027K1920Q	HMGB2	1.82×10^{-1}	RZPDp9028I2212Q	PSAP	9.28×10^{-4}
RZPDp9027B1912Q	VIMP	2.74×10^{-1}	RZPDp9028M1019Q	TRIM78P	7.68×10^{-4}
RZPDp9028A0521Q	GOLGA7	7.65×10^{-1}	RZPDp9028M219Q	ZEB1	6.39×10^{-4}
RZPDp9028J131Q	SFN	9.72×10^{-1}	RZPDp9028I2112Q	RCSD1	5.71×10^{-4}
RZPDp9028K1319Q	SRPR	9.07×10^{-1}	RZPDp9028M099Q	SRRM2	3.32×10^{-4}
RZPDp9028F0420Q	FAM21A/FAM21C	8.46×10^{-1}	RZPDp9028O1020Q	UQCRC1	2.93×10^{-4}
RZPDp9027G075Q	GLOD4	5.81×10^{-1}	RZPDp9028D249Q	MDFIC	2.03×10^{-4}
RZPDp9028A133Q	WNK2	3.42×10^{-1}	RZPDp9028M0811Q	ADH5	1.94×10^{-4}
RZPDp9027K199Q	KCTD15	2.76×10^{-1}	RZPDp9028D1211Q	SREBF2	1.06×10^{-4}
RZPDp9027K1910Q	PPP1CA	2.50×10^{-1}	RZPDp9028D213Q	MEGF6	6.79×10^{-5}
RZPDp9027P138Q	MC1R	2.46×10^{-1}	RZPDp9028F0114Q	TP53	6.65×10^{-5}
RZPDp9028N196Q	ALDOA	2.00×10^{-1}	RZPDp9028A2418Q	PSMC4	3.29×10^{-5}
RZPDp9028L013Q	CLDN5	1.58×10^{-1}	RZPDp9028E218Q	PCBP1	2.58×10^{-5}
RZPDp9028A1721Q	BAZ1A	1.41×10^{-1}	RZPDp9028I2111Q	ZC3H13	7.50×10^{-6}
RZPDp9027P046Q	FGFR3	1.40×10^{-1}	RZPDp9028E1012Q	SUMO1P3	5.30×10^{-6}
RZPDp9028C0813Q	MED20	1.37×10^{-1}	RZPDp9028M236Q	CD81	5.00×10^{-6}

Table S2. Classifier list established with class prediction using ComBat-adjusted data and 25 greedy pairs using all cases (n = 100) *versus* all controls (n = 100). For class prediction results compare Table 5.

UniqueID	Gene Symbol	*p*-value
RZPDp9027C1510Q	CNPPD1	$< 1 \times 10^{-7}$
RZPDp9027H1613Q	NONO	$< 1 \times 10^{-7}$
RZPDp9027L0419Q	KIF5A	$< 1 \times 10^{-7}$
RZPDp9027J0410Q	U2AF1	1.00×10^{-7}
RZPDp9027A0816Q	SPTBN4	1.00×10^{-7}
RZPDp9027H1611Q	WBP11	2.00×10^{-7}
RZPDp9027M0416Q	PRKAG1	4.00×10^{-7}
RZPDp9027N199Q	MRPL10	5.00×10^{-7}
RZPDp9027N1810Q	BRD2	1.10×10^{-6}
RZPDp9027N047Q	TANK	1.30×10^{-6}
RZPDp9028F183Q	SIRT7	1.40×10^{-6}
RZPDp9027F0610Q	WHSC2	1.40×10^{-6}
RZPDp9027J1913Q	RPL18	1.60×10^{-6}
RZPDp9027G0410Q	PIN1	2.40×10^{-6}
RZPDp9027K065Q	CAP1	2.70×10^{-6}
RZPDp9027F079Q	ZNF638	3.60×10^{-6}
RZPDp9028P1819Q	XPO4	4.40×10^{-6}
RZPDp9027J0512Q	SGK2	5.00×10^{-6}
RZPDp9027L1612Q	EDC4	5.20×10^{-6}
RZPDp9027H168Q	BCAS2	7.80×10^{-6}
RZPDp9028L0418Q	EIF3M	8.10×10^{-6}
RZPDp9027D1610Q	MARCH2	1.00×10^{-5}
RZPDp9028L1519Q	ARHGEF18	7.02×10^{-5}
RZPDp9027L045Q	FAM189B	1.00×10^{-4}
RZPDp9028L1514Q	FPGS	2.04×10^{-4}
RZPDp9027F194Q	R3HCC1	8.80×10^{-3}
RZPDp9027A1112Q	GSTM4	1.30×10^{-3}
RZPDp9027G1218Q	EPS8	1.28×10^{-3}
RZPDp9027L236Q	RPL9	8.37×10^{-4}
RZPDp9027O0115Q	OTUD1	5.74×10^{-4}
RZPDp9027A2412Q	POLR2B	3.16×10^{-4}
RZPDp9028I095Q	C12orf32	7.67×10^{-5}
RZPDp9027M114Q	AGT	2.04×10^{-5}
RZPDp9028D213Q	MEGF6	1.77×10^{-5}
RZPDp9028A2418Q	PSMC4	9.40×10^{-6}
RZPDp9028I2111Q	ZC3H13	8.40×10^{-6}
RZPDp9028G0813Q	IMPDH2	5.70×10^{-6}
RZPDp9027A1812Q	RRP9	5.70×10^{-6}
RZPDp9028E1012Q	SUMO1P3	5.60×10^{-6}
RZPDp9028M126Q	ZNF629	4.80×10^{-6}
RZPDp9028H1213Q	RABGGTB	4.20×10^{-6}
RZPDp9028M2311Q	CFDP1	4.10×10^{-6}
RZPDp9027A093Q	SRSF3	3.60×10^{-6}
RZPDp9028E098Q	CLIP1	3.40×10^{-6}
RZPDp9028I2310Q	ZNFX1	3.20×10^{-6}
RZPDp9028M236Q	CD81	2.50×10^{-6}
RZPDp9027M1013Q	WDR73	1.10×10^{-6}
RZPDp9027E239Q	OCIAD2	8.00×10^{-7}
RZPDp9027P239Q	U2SURP	4.00×10^{-7}
RZPDp9028H1210Q	NFKB1	3.00×10^{-7}
